# The Amino Acid Alphabet and the Architecture of the Protein Sequence-Structure Map. I. Binary Alphabets

**DOI:** 10.1371/journal.pcbi.1003946

**Published:** 2014-12-04

**Authors:** Evandro Ferrada

**Affiliations:** Santa Fe Institute, Santa Fe, New Mexico, United States of America; University of Muenster, Germany

## Abstract

The correspondence between protein sequences and structures, or *sequence-structure map*, relates to fundamental aspects of structural, evolutionary and synthetic biology. The specifics of the mapping, such as the fraction of accessible sequences and structures, or the sequences' ability to fold fast, are dictated by the type of interactions between the monomers that compose the sequences. The set of possible interactions between monomers is encapsulated by the potential energy function. In this study, I explore the impact of the relative forces of the potential on the architecture of the sequence-structure map. My observations rely on simple exact models of proteins and random samples of the space of potential energy functions of binary alphabets. I adopt a graph perspective and study the distribution of viable sequences and the structures they produce, as networks of sequences connected by point mutations. I observe that the relative proportion of attractive, neutral and repulsive forces defines types of potentials, that induce sequence-structure maps of vastly different architectures. I characterize the properties underlying these differences and relate them to the structure of the potential. Among these properties are the expected number and relative distribution of sequences associated to specific structures and the diversity of structures as a function of sequence divergence. I study the types of binary potentials observed in natural amino acids and show that there is a strong bias towards only some types of potentials, a bias that seems to characterize the folding code of natural proteins. I discuss implications of these observations for the architecture of the sequence-structure map of natural proteins, the construction of random libraries of peptides, and the early evolution of the natural amino acid alphabet.

## Author Summary

If we were to design a proteome, what types and what proportion of amino acids would we use in order to optimize properties such as the diversity of sequences and structures, their robustness to mutations, or their ability to fold efficiently? Here, I use simple models to study the sequence-structure map of proteins from a design and evolutionary perspective. These models can be used to explore all sequences and structures, as a function of the types of interactions encoded by the sequence. I study the range of possible binary interactions between monomers, which include natural and artificial amino acids. The results indicate that different amino acid compositions induce vastly different sequences-structure maps. I classify and study the properties of these maps and relate their features back to the type of energy interactions. I compare these observations to the types of interactions observed in natural amino acids. My observations provide insights for our current view of the sequence-structure map of natural proteins, guiding principles for the construction of random libraries of peptides, and suggests constraints for the early evolution of the natural amino acid alphabet.

## Introduction

The implications of understanding the properties and organization of the *sequence-structure map* of proteins are broad, they range from explaining the diversity of known protein folds in the context of cellular physiology and their evolution [1], synthesize molecules of biomedical or industrial interest [2], to engineer polymers [3] and proteomes *de novo*.

From an evolutionary standpoint the relation between sequence and structure is a particular case of a more general problem known as the *genotype-phenotype map* (GP map) [4]. According to the GP map framework, protein sequences correspond to genotypes and structures to phenotypes [5]. By using a measure of distance (*e.g.* the number of point mutations necessary to transform one genotype into another), sequences can be thought as part of a space of genotypes [6]. A graph theoretic representation of genotype space provides a quantitative, unifying framework to explore different properties of the sequence-structure relation, while considering these properties on a broader evolutionary perspective. In the following, I refer to this detailed characterization of the sequence-structure map, as its *architecture*.

The study of the sequence-structure map of proteins unifies three research programs. First, the structural biologist's, seeking to understand the limits of structural diversity and its relation to sequences in the context of a universe of folds [7]. Second, the evolutionary biologist's program, focused on the role of selection versus neutral forces shaping the architecture of the map [8], [9], as well as on the nature and role of mutational mechanisms on the origin and evolution of biomolecules [10]. And third, the protein engineer and synthetic biologist's, interested on identifying regions of genotype and phenotype space, amenable to *in vitro* search and design [2].

Simple models of polymers, so called *protein lattices* or *simple exact models* (SEMs) [11] have been used extensively to explore the sequence-structure relation of proteins. These models were originally developed to study the dynamics of polymers by modeling key thermodynamic properties that govern folding [12]. They consist of short sequences (e.g. 12 to 36 mers), composed of a limited alphabet size, usually 2 to 20 monomers. Sequences are folded onto a lattice of fixed dimensionality (i.e. 2 or 3-dimensional) and geometry (e.g. square, cubic, FCC, etc). The most common SEM is the *HP model*, consisting of only 2 monomers (i.e. H, hydrophobic and P, polar). In the HP model only H-H contacts contribute to the stability of the conformation [12]. Their main limitation relates to *finite size effects*. That is, artifacts arising as a consequence of the model's geometry, dimensionality, and polymer length; which introduce biases on the relation between surface versus core residues and long-range interactions [13], [14]. These limitations have been proven detrimental to the study of folding kinetics and the cooperative two-state transition of globular proteins, for which the use of detailed atomistic models is advised [15].

Despite these limitations and their simplified representation of the geometric complexity of protein structures, SEMs have been instrumental in understanding a variety of aspects of protein biology [13]. They have been used to study theories and mechanisms of protein folding [13]; the distribution of sequences versus structures [16]; determinants of folding kinetics [17]; protein design [14]; recombination [18]; protein-protein interactions [19]; misfolding and aggregation [20]; the study of energy functions and their performance [21]; comparative modeling [22]; neutral networks and innovation [23] and protein evolution [11], [24], among others. In contrast to the study of natural proteins, SEMs can be used to fully characterize the sequence-structure map, that is, the relation of all possible sequences to all possible structures. Their strength relies on the characterization of large number of sequences and conformations, and therefore on the study of phenomena for which the statistics predominate over the details of folding [25].

A first relevant property of the sequence-structure map of proteins is that not all possible sequences are equally likely to encode a structure. Different criteria has been employed to decide on the propensity of a sequence to fold. In general, these criteria consider key thermodynamic determinants that distinguish between the stability of a sequence across conformations. For instance, the total stability of a sequence on its native conformation (E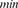) [26], the energy difference (*i.e. energy gap*) between E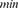 and the next stable conformation(s) [27], or the deviation of E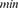 from the ensemble of all possible conformations (*i.e. foldability* [28], see below).

Although all these criteria are approximations to the propensity of a sequence to fold, the *degeneracy* (*g*) of a sequence have proven a useful proxy to distinguish between foldable and random polypeptides. *Degeneracy* corresponds to the total number of conformations that a given sequence stabilizes at its minimum observed energy. Under this criterion, a sequence is considered foldable if it is *non-degenerate* (*i.e. g* = 1).

In the case of SEMs, the stability of a protein sequence, folded onto a given conformation, can be approximated by the strength of the interactions between non-adjacent residues along the peptide chain. These interactions are encapsulated by a *potential energy function*, or simply, *potential*.

The derivation and performance of potentials have been the subject of a long research tradition [29]. The most successful potentials are the result of statistical approximations that derive propensities of interactions between monomers from a large set of protein crystal structures (*i.e. knowledge-based* or *statistical* potentials). The physical interpretation of these forms of energy functions, however, remains a subject of debate [30]. One of the reasons is that, statistical potentials ignore much of the details of the interactions between residues in proteins. A major distinction between statistical potentials is the use of different *reference states*. The study of a diverse set of statistical energy functions derived using different reference states shows that most of them describe two putative stages during folding [30]. On the one hand, some potentials characterize the hydrophobic collapse of globular proteins [29], [31]–[34]. On the other hand, they might reflect subtle differences among residue interactions at the native or near-native state [29], [35]–[37]. Similar to the approximation employed by SEMs, statistical potentials have been successfully used to score the stability of protein crystal structures, and protein models, by only considering the pairwise interactions of amino acids [38].

Similar to the concept of degeneracy, one may consider the fraction of conformational space encoded by non-degenerate sequences, or *encodability* [39]. Both, non-degeneracy and encodability are closely related properties. They depend on the amino acid alphabet size and composition, which in turn defines the potential.

In 1996, Chan and Dill [39], studied the impact of properties of the potential on degeneracy and encodability. They explored the role of repulsive interactions and correlations between energy values on well-known binary potentials and showed that the nature of the potential affects the sequence-structure map and, in doing so, it is as important as the size of the alphabet. Specifically, they studied the HP model and a modified version, the AB model; and showed that repulsive interactions reduce the average sequence degeneracy and consequently, increase the fraction of foldable sequences and encodable structures.

While non-degeneracy and encodability describe the fraction of accessible sequences and structures, a full description of the sequence-structure map should also account for the relative use and distribution of sequences and structures in genotype and phenotype spaces. The language of graphs has been used to represent and study the distribution of sequences in genotype space [6], [40]. According to this paradigm, groups of non-degenerate sequences that fold onto the same structure and can be connected to each other by single point mutations, are known as *neutral networks* [5]. The size of neutral networks has consequences for the evolution of phenotypes. Arguably, sequences that are part of a large neutral network can undergo a considerable number of mutations while still preserving their phenotype. These phenotypes are found more frequently by a random search on genotype space and because of their robustness to mutations, represent good candidates for protein design experiments [41].

Following Maynard-Smith's concept of protein space [6], Lipman and Wilbur used the HP model to explore the existence and general statistics of neutral networks [40]. They observed that sequences folding onto the same conformation, map to nearby regions of genotype space and can be reached from various mutational paths. Subsequent studies, inspired by analysis of the RNA GP map, used SEMs to analyze the distribution of neutral networks in sequence space. These studies showed that neutral networks of the HP model distribute on isolated regions of genotype space, with unfrequent mutational paths between networks [42].

Other studies have explored the distribution of genotypes' stabilities in neutral networks [42], [43]. They showed that neutral networks have a *funnel-like* organization, where the most stable sequence usually corresponds to the network's ‘average’, or *consensus* sequence. The relation between structural stability and consensus sequence has been explored experimentally [44]. These authors have also compared the neutral networks between the HP and AB models. They demonstrated that features of the potential impact the number, size and longest paths of these networks [25], [43].

While sequences of a neutral network use nearby regions of genotype space, sequences that preserve the same phenotype may also occupy divergent regions of genotype space. These type of sequences, that belong to disconnected neutral networks in genotype space, are called *neutral set* [42]. Neutral sets are usually characterized by their size, in number of sequences, or *designability* [16].

Li et al (1996) used two and three-dimensional SEMs to show that designability distribute slightly less than exponential over conformations [16]. In other words, most conformations are associated to a single or few sequences, while few conformations use a large fraction of the available space of genotypes. At the time, this was a remarkable observation, because it recovered the biased distribution of the number of sequences per structure observed from very sparse natural samples [45]. Since then, two related hypothesis have been proposed to explain the origin of the vast differences on the designability of protein structures.

One hypothesis relies on the requirement of structural stability [46]. Structural stability correlates closely with the total number of contacts of a conformation (or *compactness*). Since the contribution to the total energy of a sequence folded onto a conformation is given by the number of contacts between residues, the larger the number of contacts, the more stabilized a conformation can get and consequently, the larger the sequence variability. In other words, compact conformations are intrinsically designable.

A second hypothesis concentrates on the propensity of sequences to fold fast [27]. Folding can be seen as a competition of a sequence for conformations. The diversity and stability of conformations surrounding the native structure is a measure of a sequence's ability to fold efficiently. This property is called *foldability* [28]. Different theoretical formalisms have been proposed to quantify it. Intuitively, these formalisms consider the *energy gap*, or difference in stability between the sequence folded onto its native structure and the stability at the next(s) most stable conformation(s). In other words, foldability is a measure of the steepness of the energy landscape surrounding the native structure.

The concept of *foldability* does not aim to provide mechanistic details on the protein folding path, but simply identify important energetic features that distinguish natural proteins from random polymers [47]. Similar concepts rely on the same principle, such as the comparison of conditions for folding versus the conditions for chain collapse [48], or the principle of minimal frustration [27]. Theory based mainly on the random energy model and extensive simulation studies, have demonstrated the practical value of this idea. Other studies have also shown that this criterion alone, does not fully address the degrees of kinetic and thermodynamics complexity of natural proteins [15]. However, in the context of simple exact models, as it been studied before, the concept of foldability remains a good approximation as to how protein-like a polymer is [49], and as a requirement for protein design [14].

Designability and foldability capture different aspects of the sequence and structural constraints imposed on folding. Govidarajan and Goldstein showed that conformations have different foldabilities and that optimally foldable conformations are also highly designable [28], [47]. Buchler and Goldstein [50] used 25 mer, a two-dimensional, maximally compact SEM, to explore the distribution of designabilities under a range of amino acid alphabets and foldability requirements. They observed that, under these large variety of parameters, the distribution of designabilities remain strongly biased across conformations. This finding let them to suggest that designability is a general property of the protein GP map. The distribution of designability across structures, however, is highly dependent on the size of the amino acids alphabet, as is the identity of the most designable structures [50]. From an evolutionary standpoint the designability of a network of sequences, as well as their foldability, are important determinants of the *mutational robustness* of a phenotype [51].

In addition to the properties of isolated networks of sequences, a full description of the protein sequence-structure map should account for the distribution of neutral networks across genotype space relative to other networks and to the phenotypes that they map onto. Similar to the concept of designability, in revealing aspect of the mutational robustness of a phenotype, a sequence's accessibility to different phenotypes is a property of evolutionary relevance. This is because, the larger the phenotypic diversity in a neighborhood of sequence space; the larger the capacity of a sequence to innovate upon mutation [52], [53]. Because the amino acid alphabet, and therefore the potential energy function, impacts the fraction of foldable sequences and the encodability of phenotypes, arguably, it may affect the relative distribution of phenotypes respect to other phenotypes across sequence space, and consequently, impact both, the map's constraints on the accessibility to new phenotypes, as well as, its general ability to innovate through mutation.

Recent advances in the *de novo* design and synthesis of polymers [3], the synthesis and manipulation of entire chromosomes [55], as well as, the introduction of new amino acids into the genetic code [54]; has opened new perspectives and challenges that touch upon these ideas. If we were, for instance, to choose the monomers to engineer a proteome, what types and proportion of interactions would we include in order to optimize mutational robustness, the fraction of accessible genotypes and phenotypes, and/or their foldability? This question suggests a *sequence-structure map problem*, that is not concerned with the mechanisms of folding, but with predicting the architecture of the map, given the composition of the amino acid alphabet.

Similar questions exist in the field of protein design [2]. The construction of large random libraries of polypeptides used in *in vitro* search studies, would benefit of understanding what *number* and *types* of natural or *artificial* amino acids may promote sequence and
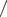
or structural diversity [56], [57].

Yet another significant area of research relates to the origin and establishment of the early genetic code [58]. What is the minimal number and types of amino acids that allow the synthesis of a primordial, protein-like sequence-structure map of proteins? [58], [59]. This is a question that has haunted a wide variety of research fields since the late 60's [60], and for which there are partial theoretical and empirical insights [14], [61]. Although a thorough exploration of the myriads of factors involved in the early evolution of the genetic code is beyond the scope of the present study, an understanding of the relation between amino acid composition and the sequence-structure map, might provide indirect evidence on fundamental constraints that affected the establishment of the primordial amino acid alphabet of proteins.

In this work, I study the impact of the potential energy function on the architecture of the protein sequence-structure map. I use SEMs, sample the space of possible binary potentials, and study the properties of the maps they induce. I analyse properties such as non-degeneracy, encodability, designability and foldability, the connectivity and relative distribution of neutral networks, as well as the overall phenotypic diversity of the sequence-structure maps induced by these potentials. I study the types of binary potentials present in natural amino acids and compare them to a random sample of the space of potentials. A detailed exploration of these properties may first, provide an alternative view of the sequence-structure map of natural proteins; second, help to explore the limits imposed by the architecture of the sequence-structure map on the evolution of proteins; and finally, may provide insights on the construction of random libraries of peptides and the large-scale design of sequence-structure maps with desired properties.

## Models

### Genotype space as a graph

A simple exact model (SEM) consists of three main parameters: sequence length (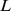), monomer alphabet (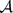) and the potential (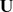). Genotype space (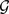), is composed of 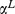 sequences. Where 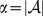. (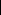 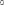 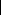, is the *cardinality* of the set 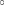). The dimension of 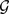, 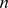, is defined as the total number of single point mutant neighbors of a given sequence, as: 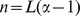. For 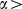
2, 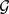 is called a *generalized* hypercube (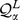). A sequence 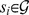, is composed of 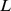 monomers 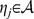. A hamming distance metric, 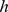, over 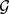, defines a *n*-cube or hypercube, where 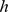
(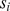,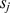), corresponds to the number of point mutations needed to transform genotype 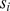 into 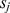 [62]. Similarly, the space of phenotypes, 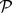, corresponds to the set of all possible conformations. The enumerable conformational space is independent of 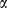 and growths exponentially as a function of 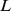.

The *potential energy function*, 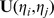, specifies the energy associated to the interaction between monomers 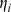 and 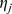. The total stability (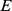) of a sequence 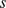 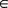 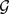, folded onto conformation 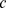 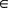 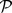, is defined as:
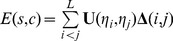
(1)


The function 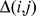, adopts a value of 1 if monomers at positions 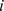 and 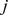 are in contact and non-adjacent along the chain, 0 otherwise. The degeneracy (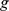) of sequence 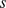 corresponds to the number of conformations adopted at 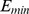 (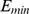 = min(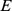)). According to the *thermodynamic hypothesis* of protein folding, a sequence 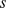 folds onto a conformation 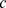 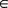 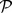, if and only if, 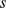 is non-degenerate on 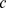 (i.e. 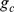 = 1). In that case, 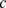 is called the *native* structure of 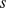.

### Genotype neighborhoods and phenotypic diversity

The *k*- *neighborhood* of a sequence 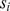 is defined as the set of sequences at a hamming distance equal or lower than *k*, respect to 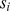 (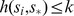). The number of sequences of a *k*-neighborhood increases as 
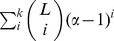
. For 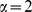 and 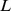 = 18; 1, 3, and 5-neighborhood contain 18; 987; and 12,615 sequences, respectively.

In order to quantify the relative distribution of phenotypes across sequence space, I consider the *phenotypic diversity* of a *k*-neighborhood centered at a sequence 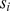 (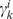). 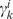 is simply defined as the set of phenotypes encoded by sequences in the *k*-neighborhood of 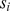. 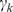 for small *k* values, informs on the fraction of immediate accessible phenotypes, those expected to be available after few point mutations; whereas larger *k* values, tell us about the overall diversity of phenotypes across sequence space.

### Sequences, networks and components

By applying Eq. 1 over all sequences in 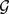, a given potential 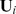, induces the folding (i.e. *mapping*) of a set of non-degenerate sequences (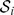), which represents a fraction of genotype space (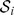 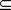 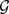); into the set 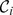, a fraction of phenotype space (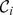 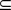 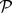). We say that 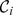 is the *accessible conformational space* induced by the potential 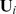 on 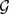. The total fraction of non-degenerate sequences induced by 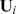, is called *non*- *degeneracy* (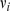 = 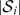 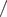 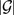 = 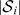 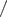 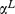). Similarly, *encodability* can be defined as: 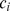 = 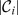 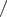 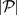.

**Figure 1:**
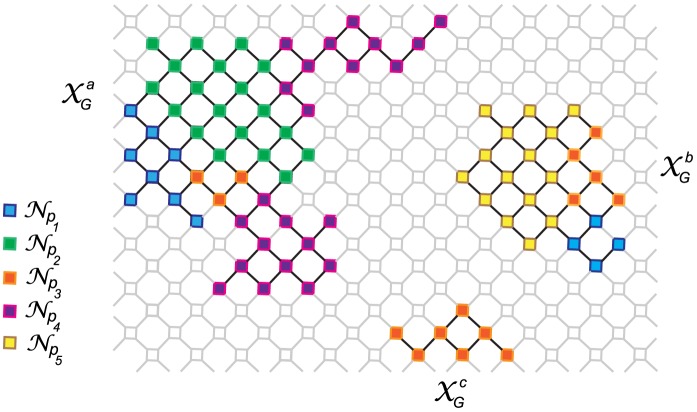
A two-dimensional caricature of a genotype network (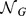). Sequences are represented as nodes. Edges are drawn between sequences that differ in 1 mutation. Degenerate sequences (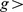
1) are in grey, open squares. In this example, 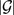 = 192 nodes; 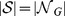 = 84 nodes; 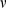 = 0.43. 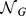 is composed of 3 genotype components (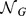 = (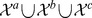)). Top left, 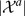 = 53; top right, 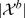 = 24; bottom, 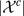 = 7. Non-degenerate sequences in this example, fold onto 5 phenotypes represented by the neutral sets in colors blue, 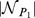 = 12; green, 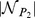 = 19; orange, 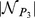 = 15; magenta, 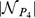 = 23; and yellow, 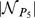 = 15. Genotype components 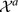 and 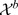 are composed of more than 1 neutral network. 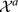 = {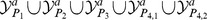} and 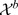 = {
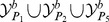
}. Phenotype 1 (blue) can be found in genotype components 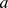 and 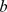: 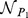 = {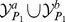}. Phenotype 2 (orange) can be found in all three genotype components: 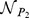 = {
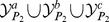
}. Genotype component 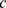, is also a neutral network: 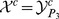.

The non-degenerate fraction of sequence space induced by 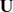, can be treated as a *network of genotypes* (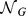) (Figure 1). Sequences are *nodes*, and *edges* are formed between pairs of sequences that differ in one point mutation (*h*(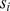,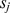) = 1). When two nodes in 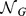 can be connected by a series of single point mutations, we say there is a *mutational path* (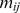) between them. The diameter of a graph corresponds to its largest 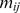.

A connected component of a genotype network, or *genotype component* (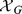), is a subset of nodes in 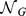, for which there is at least one 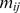 between every possible pair of sequences (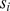,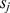). A genotype network can be composed of one or more than one genotype component (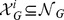); and the total number of sequences in the network is the sum of the number of sequences in each component (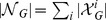). Note that 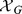 represents the set of genotype components, 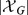 represents the number of genotype components, whereas 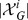, the size, in number of sequences, of genotype component 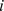. For instance, 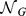 in Figure 1, is composed of three 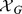: 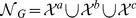. (I drop the subscript *G*, since all genotype components are necessarily part of 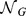).

The distinction of genotypes according to the phenotypes they map onto, induces subgraphs, whose properties have important consequences for the architecture of the map and can be characterized quantitatively in terms of the statistics of their expected size, diameter and distances. Sequences that fold onto the same phenotype are called *neutral sets* (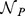) and are, by definition, subsets of the genotype network (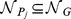). Note that the number of 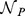 is equivalent to the number of accessible phenotypes (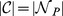). For instance, in Figure 1, 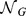 is composed of 5 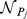, represented by different colors.

Sequences are known to distribute heterogeneously over conformations and this property of a phenotype, traditionally called *designability* (C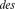) [16], has important implications for evolution and design. The designability of a phenotype *j* is equivalent to the size, in terms of number of sequences, of the neutral set associated to phenotype *j* (C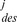 = 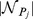).

As in the case of 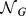, a neutral set (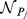) can also be composed of more than one connected component. These connected subsets of non-degenerate sequences, that map to the same phenotype, are called *neutral networks* (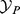). Note the subscript distinction. 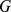 refers to genotype, as in genotype network (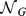) and genotype component (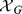). 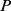 refers to phenotype. However, instead of using *phenotype network* (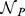) and *phenotype component* (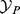), I stick to the terms traditionally used in the literature: *neutral sets* and *neutral networks*, respectively [5], [42].

A neutral set can be composed of more than one neutral network (
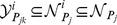
). 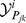 refers to the 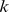 neutral network in the neutral set of phenotype 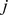 (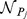); and genotype component 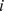. As is the case of 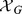, all pairs of sequences in 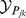 are connected by at least one mutational path. For instance, Figure 1, shows 9 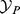. The largest genotype component in 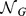 (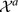), is composed of 4 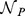 and 5 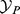.

Similar to the idea of designability (C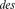), here I define a network's *neutrality* as the size, in number of sequences, of a neutral network, as: C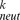 = 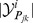. Whereas the *neutrality* of a single sequence 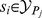 (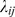), is defined as the fraction of mutants in the 1-neighborhood of 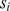, that are part of 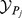.

A genotype component (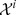) in 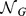, can be composed of more than one neutral network (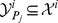). But, note that not all neutral networks (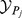) of a given neutral set (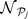), are necessarily part of the same genotype component (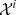). Sequences in 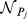 are the sum of sequences in neutral networks (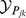) that can be part of different genotype components: 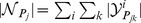. For instance, 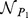 (Fig. 1, in blue), can be expressed as 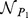 = (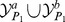). (See Table S2 in Text S1 for a summary of symbols and abbreviations).

### Ideal and excess parts of a potential

**Figure 2:**
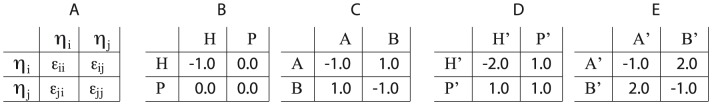
Potentials for the canonical HP and AB models. (A) General structure of a binary potential, composed of monomers 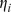 and 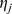. Potentials of the HP model (B), AB model (C), HP shifted (D), AB shifted (E).

A binary potential can be represented as a vector composed of 3 values, that describe 2 types of interactions (Figure 2A). First, those between the same type of monomers, or *homomonomeric* (*i.e.* 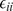, 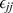); and second, the *heteromonomeric* interaction (*i.e.* 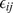). The heteromonomeric interaction of a binary potential can be decomposed into *ideal* and *excess* parts [12], [30]. These parts describe the extent to which the potential favors two different hypothetical stages of the folding process. The ideal part represents an heteromonomeric interaction as in an ideal liquid. That is, as if there was no energetic contribution by the heteromonomeric interaction, and therefore it could just be approximated by the arithmetic mean of their homomonomeric 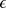 values, as: 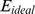 = (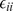
+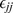)/2. In contrast, the excess part (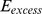 = 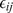 - 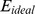), aims to capture the contribution of the heteromonomeric interaction, and describe the extent to which the native conformation differs from an ideal mixture of amino acids, its *additivity* (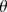). Here, I quantify the additivity of a given potential as: 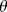 = [E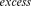/E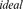]+1 = 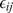/E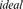.

### The L18 model

In this study I use a two-dimensional SEM of sequence length 18 mer. In the following I refer to this model as L18. The motivations for using this model, are fourfold. First, L18 represents a good compromise in relation to the number of sequences versus the number of conformations. Second, inspired by globular proteins, some previous studies assume that foldable sequences must adopt a maximum number of contacts. Because the restriction of phenotype space to maximally compact conformations introduces artifacts, as inflated values of designabilities [50], here I consider sequences folding onto any possible conformation, as long as, the thermodynamic criterion is met. Third, compared to three-dimension, two-dimension SEM show a surface-core ratio more similar to natural proteins [13]. Finally, the L18 model has been extensively used to evaluate different alphabets and potentials [42], [43], which will allows us to compare our results to previous findings.

In the case of L18, 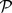 is composed of 5,808,335 total conformations, and 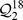, of 262,144 sequences. Because the energy of a sequence folded onto a given conformation is here approximated by the contact of non-adjacent monomers along the chain, conformations in a lattice are usually represented as *contact sets*, a binary symmetric L by L matrix that describes the interactions of non-adjacent monomers [63]. Due to the larger degrees of freedom of conformations with few contacts, different conformations may correspond to the same contact set. The total 5,808,335 conformations of the L18 model, can be described by 170,670 non-redundant contact sets. Only 77,635 out of the 170,670 contact sets, are unique (ie. each one of them correspond to a single conformation) and therefore *potentially encodable* (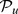) under the thermodynamic hypothesis criterion [39]. The accessible conformational space of a sequence-structure map, represents a subset of the uniquely encodable set of conformations (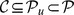).

### Foldability

A sequence's *foldability* (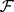), is mathematically described as the deviation of the energy minimum from the energy distribution of the ensemble of all possible conformations in 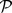 [50]:
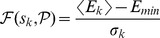
(2)

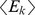
 is the expected stability of sequence 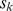, over all possible conformations in the ensemble; 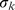, the standard deviation over the same distribution; and 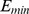, the minimum observed energy of 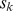 folded onto 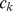. A more negative 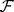 value describes a steeper folding funnel and therefore protein-like behavior.

## Results

In order to explore the impact of the potential on the architecture of the sequence-structure map of natural proteins, I concentrate on the L18 model and binary alphabets. The computational tractability of this model allows us to study exact statistics of a large sample of potentials.

### Types of binary potentials and the space of phenotypes

The potential of a binary alphabet is described by three values: 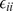, 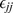 and 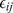 (with 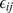 = 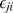, see Figure 2A). 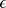 values are real numbers. If negative, they correspond to *attractive* interactions. If positive, *repulsive*. *Neutral* interactions (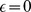) do not contribute to stability. Because of the symmetry (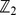) of the cube (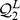), homomonomeric interactions (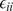 and 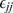) are interchangeable. In other words, if all 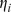 monomers were exchanged by 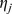 monomers, properties of genotype space would remain the same.

The first protein lattice model ever studied was the HP model [12]. It is composed of two types of amino acids, polar (P) and hydrophobic (H). The potential is detailed in Fig. 2B. Only homomonomeric hydrophobic interactions (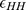) contribute to the stability of a folded sequence.

An alternative to the HP model, the AB model, was introduced in order to explore the impact of the potential on protein design [64]. The AB potential introduces equivalent interactions between homomonomers (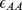 = 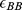 = −1.0) and a repulsive interaction (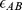 = 1.0), (Fig. 2C). The HP and AB potentials have been modified (the so called *shifted* potentials) to study explicitly the impact of repulsive interactions [39]. Figures 2D and 2E show the shifted versions of HP and AB potentials. I refer to these 4 potentials as *canonical*.

In order to investigate the impact of the potential on the sequence-structure map, I begin our analysis by sampling the *space* of possible binary potentials, with 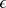 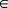 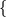
−1.00, −0.75, −0.50, −0.25, 0.00, 0.25, 0.50, 0.75, 1.00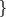; of which, canonical potentials are a small subset. Since a binary potential is composed of three 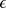 values, our sample produces a total of 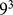 possible 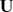. From these total possible combinations one must ignore potentials with no relative favorable interactions (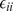 = 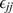 = 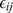), potentials with only repulsive or neutral interactions (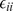, 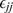, 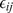 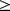 0.0), scaled potentials of the form 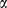 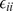, 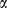 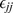, 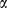 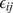 (with 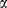 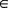 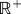), take into account the symmetry at homomonomeric interactions (i.e. 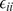,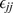,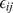 = 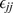,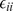,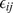), and, the symmetry between homo versus heteromonomeric interactions (*i.e.* if 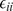 = 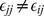; then, 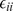, 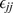, 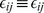, 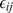, 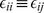, 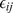, 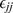). These considerations result on a total of 245 potentials.

**Figure 3:**
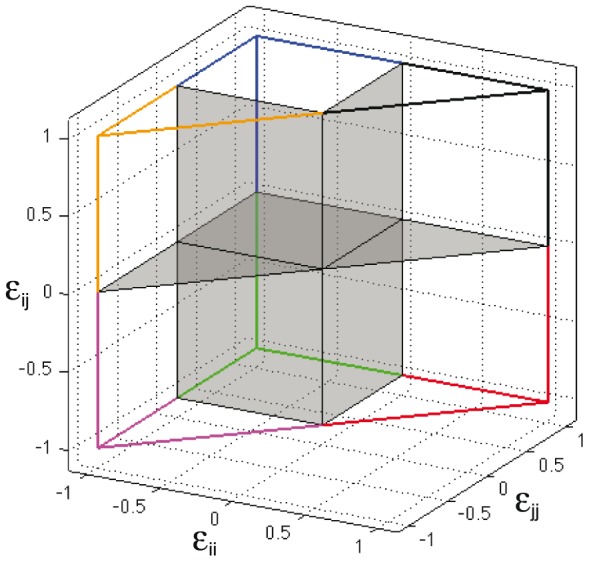
Binary potentials as vectors of 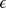 values. Figure show a graphic representation of the 7 types of potentials described in Table 1. These potentials (type I-V, VII), correspond to the 6 non-redundant octants in the 3d representation of 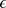 coordinates. Potentials type VI, those with at least one 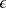 = 0.0, are represented by grey planes between octants.

Potentials can be represented as vectors. Due to the symmetry of the cube (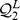), half of the space contains all possible non-redundant binary potentials. As suggested by previous studies, many properties of the potential energy function are determined by the proportion of repulsive, attractive and neutral interactions [39], [50]. I use this criterion to distinguish among 7 types of potentials, that correspond to the 6 non-redundant octants in the 3d 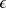-coordinates representation, plus any potential with at least one 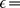 0 (Table 1, Figure 3). The octant in black (Fig. 3), that corresponds to all-repulsive interactions (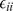, 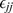, 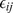 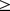 0.0); is defined as potential type VII and, by definition, does not stabilize any conformation (see Eq. 1). The 245 potentials described above are an homogeneous sample from this space.

**Table 1:** Type of potentials for binary alphabets.

Type	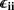	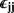	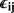	Color code
*I*	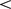 0.0	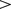 0.0	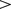 0.0	Blue
*II*	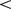 0.0	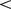 0.0	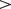 0.0	Orange
*III*	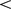 0.0	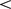 0.0	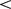 0.0	Magenta
*IV*	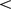 0.0	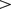 0.0	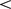 0.0	Green
*V*	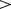 0.0	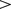 0.0	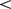 0.0	Red
*VI*	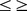 0.0	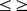 0.0	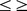 0.0	Grey
*VII*	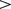 0.0	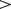 0.0	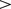 0.0	Black

For each of the 245 potentials I proceed as follows. I enumerate all possible sequences. I fold each sequence onto every contact set and calculate its stability and foldability using equation 1 and 2, respectively. (The raw data of the 245 sequence-structure maps studied here, is available at: www.santafe.edu/ 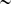
eferrada, see Table S1 in Text S1.).

In order to compare different potentials and their impact on properties of the sequence-structure map, I use *hierarchical clustering* (see Supplementary Methods). The *Jaccard similarity index*, between the sets 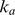 and 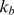 (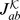), (with 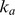, 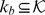; 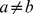), is defined as: 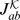 = 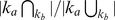. 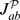, measures the similarity between the sets of conformations 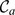 and 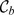 (with 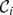 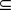 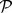), induced by the potentials 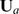 and 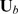, respectively. Similarly, 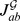, compares sets of sequences 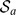 and 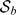 (with 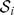 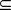 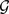) (see Supplementary Methods).

**Figure 4:**
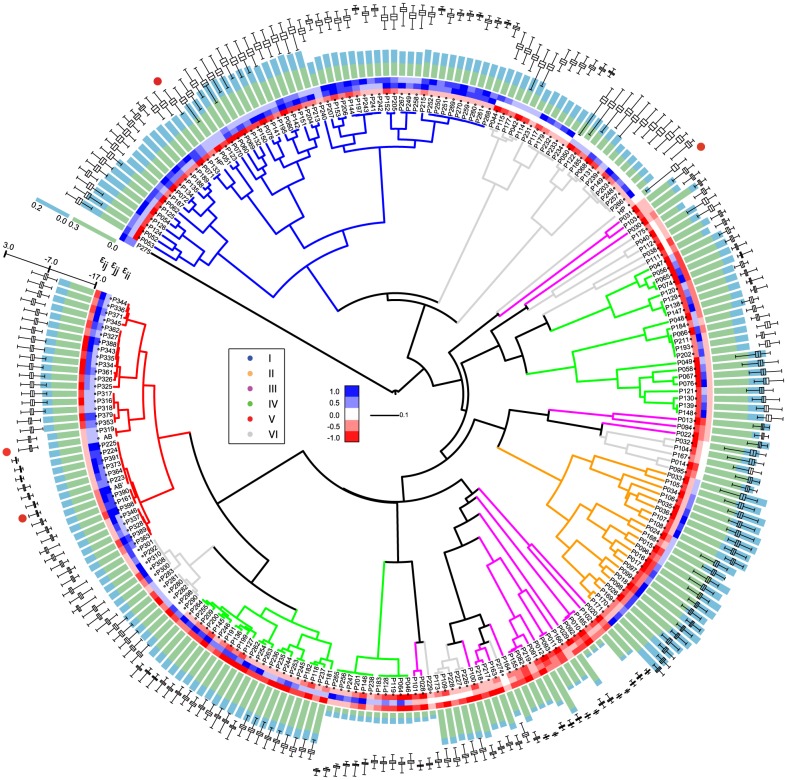
Hierarchical clustering of phenotype spaces generated by the sequence-structure maps of binary potentials. Potentials are sampled by considering 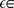 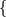
−1.00, −0.75, −0.50, −0.25, 0.00, 0.25, 0.50, 0.75, 1.00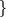 (see main text and Table S1 in Text S1). Hierarchical clustering was carried out using similarity measure 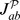 and the group-average method. 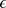 values of each potential are specified on a color scale at the branches' tips, with 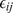 specified by the outermost value. Branches are colored according to the 7 different potentials described in Fig. 3 (see also main text and Table 1). Green and blue stacked bars following the color-coded potentials, correspond to non-degeneracy and encodability, respectively. Boxplots, in black, represent the distribution of foldability values over non-degenerate genotypes for each map. Canonical potentials are the HP and AB models and their shifted versions (Fig. 2). They are highlighted with red dots.

Figure 4 presents a hierarchical clustering of phenotype space based on 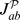 (and 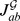), for all possible pair combinations of binary potentials 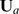 and 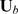 (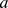, 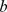 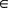 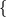
1,…, 245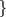). Here I arbitrarily choose to focus on 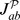, however, similar conclusions arise from the analysis of the Jaccard index on genotype space (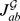) (Figure S1). Each tip of the tree represents an independent sequence-structure map. Maps that cluster closely in this tree have similar sets of accessible phenotypes (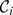), that is, 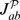 values close to 1.0. 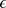 values that compose each potential are specified on a color scale at the branches' tips, with 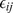, specified at the outermost value. Branches are colored according to the potential, as described above (Table 1, Figure 3). Green and blue stacked bars following the color-coded potentials, correspond to non-degeneracy and encodability values, respectively. Boxplots, in black, represent the distribution of foldability over all non-degenerate sequences of each map.

A first observation from Figure 4 is the impact of the potential on non-degeneracy, encodability and foldability, as well as the overall consistency of these properties across potentials with similar 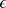 values. The potential can induce considerable differences in non-degeneracy and foldability. Confront, for instance, potentials type IV and potentials type II (green and orange branches, respectively). A similar observation applies in the case of clustering based on 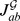. In both cases, results are independent of the clustering method (Fig. S2, and Supplementary Methods).

A second general observation regards the abrupt changes in the use of phenotype space between some of the maps with potentials of the same type. While potentials type I, II and V (blue, orange and red branches, respectively) are highly clustered, potentials type III and IV (magenta and green branches, respectively), distribute across different clusters.

Figure 4 also reveals that canonical potentials are part of a larger family of potentials, which represent only 3 out of the 7 different types described above (Table 1; Fig. 3 and 4). Most notably, other combinations of 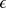 values, in particular, potentials type I and II; induce sequence-structure maps that are as protein-like as the HP model (see below, section Foldability). Moreover, potentials that induce similar fractions of sequences and structures, present considerable variation in their average foldability.

I now turn to a closer look at these differences.

### Non-degeneracy and encodability

**Figure 5:**
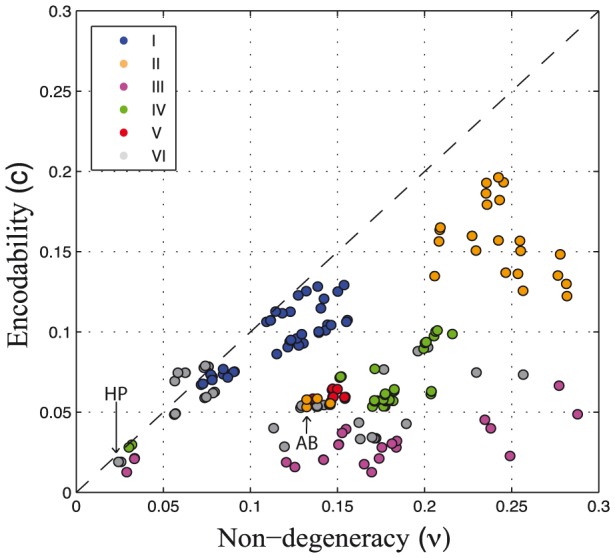
Association between non-degeneracy and encodability. For each potential sampled in this study, the plot shows non-degeneracy (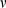) versus encodability (*c*). Non-degeneracy corresponds to the fraction of genotype space that yields viable sequences; encodability, to the fraction of accessible conformations (see *Models*). Colors match the potentials types described in Fig. 3 and Table 1.

Non-degeneracy (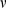) is the fraction of genotype space that yields viable, folding sequences. It ranges from 2 to 28% across maps generated by the binary potentials sampled in this study (green bars in Fig. 4). Similarly, encodability (*c*), the fraction of accessible phenotypes, varies from 1 to 19% (Fig. 4, blue bars). Both, 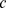 and 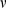 vary considerably across types of potentials (Fig. S3).


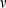
 and 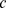 are not independent and overall, correlate positively. Their association, however, depends on the type of potential (Fig. 5). In the case of potentials type I, II and IV, an increase in 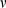 leads to larger *c* values. Potentials type III, however, preserve similar *c* despite large variation in 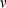. With the exception of few potentials type I and VI, maps induced by binary potentials, use a larger fraction of sequence than structure space (dashed line, Figure 5).

Two main features of the potential account for 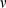 and *c*. First, low negative values of 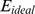, that is, average attractive homomonomeric interactions (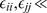
0.0), promote both increasing 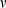 and 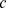 (see Fig. S4). The lowest values of 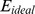 are observed in the case of potentials types II and III. Second, positive 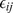 values seem to be sufficient to promote 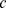, but not 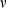 (Fig. S4 and S5). Potentials type I and II are the only potentials with positive 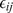 values. They present encodabilities that are on average one order of magnitude larger than the rest of the potentials sampled in this study.

These two features provide some intuition as to *why* potentials type II and III reach large values of 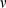, but only type II present also large values of *c* (Fig. 5); whereas potentials type V show low and conserved values of 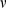 and 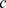. The 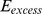 component of the potential does account for both 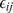 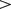 0.0 and 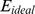 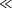 0.0. Therefore, 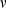 and *c* are expected to correlate positively with 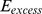 (Fig. S4B, S4D).

As observed before, repulsive interactions reduce the average sequence degeneracy, increasing 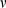 and 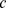 [39]. However, our analysis of a large sample of potentials shows that not any type of repulsive interaction possess this effect, but only the heteromonomeric component of the potential, and that the effect is favored in the context of overall attractive 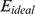.

Most notably, these observations suggest that, by controlling for the components of the potential, both, the fraction of sequence and structures can be increased and furthermore, optimized independently of one another. For instance, the average number of sequences per conformation (i.e. *designability*) can be optimized by increasing 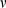 while keeping *c* constant, as is the case of potentials type III (i.e. increasing attractive interactions in both, homo and heteromonomeric components, Fig. 3 and 5).

### The use of sequence and structure space

Although 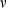 can be seen as the probability of finding a viable sequence, the distribution of sequences in genotype space is not uniform, and depends on their monomer composition.

**Figure 6:**
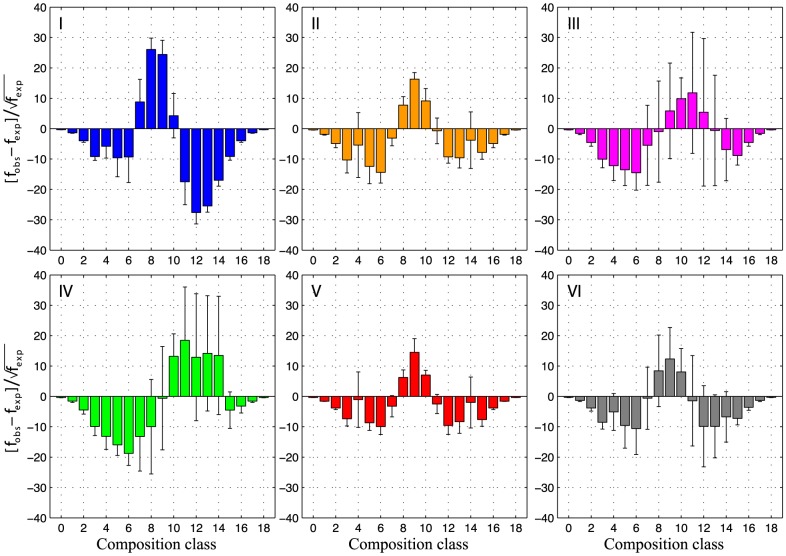
Observed versus expected compositional classes of potentials types I to VI. Compositional classes correspond to set of sequences with a given fraction of 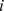 and 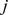 monomers. A given compositional class contains 18-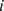 monomers type 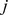. Expected number of sequences per compositional class are estimated by sampling, for a given potential 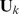, 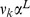 random sequences from genotype space. Error bars represent one standard deviation from the mean. Colors code each potential type according to Fig. 3 and Table 1.

Sequences can be classified according to their composition into classes. Compositional classes correspond to the frequency of the relative fraction of monomers across non-degenerate sequences induced by a given potential. In the case of maps composed of binary potentials, compositional classes distribute binomially. If all 2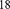 sequences in the L18 model were non-degenerate, there would be 19 compositional classes, ranging from the unique two sequences composed of only one of the two monomer types (compositional classes 0 and 18 in Fig. 6; with 0 and 100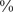 of monomer 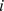, respectively) to 48,620 sequences composed of 50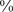 of each monomer (compositional class 9).

In order to study the distribution of non-degenerate sequences across genotype space, I compare observed versus expected frequencies of different compositional classes. Expected compositional classes are estimated for a given potential 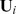, by sampling 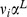 random sequences from genotype space, assuming that every sequence is equally likely to be non-degenerate.

Potentials present considerable biases toward certain compositional classes (Fig. 6). In particular, genotype spaces of potentials type I are enriched in 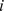 monomers, with compositional classes near 61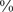. In contrast, potentials type IV show significant deviations toward 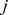 monomers. In addition, consistent with the abrupt transitions between similar potentials (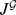, Fig. S1), potentials type III, IV and VI show considerable variation (error bars, Fig. 6).

Deviations from expected distributions can be explained by the proportion of attractive and repulsive values at homo versus heteromonomeric interactions (Fig. 3, Table 1). In the case of perfectly symmetric interactions between homo and heteromonomers, as is the case of potentials type II and V (Fig. 3, 6), there are no major deviations toward compositional classes enriched in either of the monomers. In these two cases, the diversity of repulsive and attractive interactions do not favor any compositional class. In the case of potentials type I and IV, however, one of the homomonomeric interactions breaks the symmetry of the potential, favoring the monomer that better counteracts stability respect to 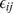, increasing the diversity of competing interactions. Thus, potentials type I favor monomers type 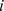 (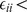
0 and 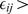
0); whereas potentials type IV, 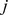 monomers (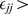
0 and 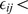
0).

Similarly, deviations in structural space can be estimated by considering the distribution of number of contacts across the conformations induced by a potential (*i.e.* compactness). The distribution of expected number of contacts can be estimated by assuming that every uniquely encodable conformation is equally likely to be accessed by non-degenerate sequences. Therefore, for a given potential (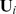), I sample 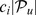 conformations and calculate their number of contacts. Where 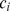, is the encodability of sequence-structure map 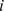, and 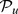, the set of uniquely encodable conformations of phenotype space (see *Models*).

All types of potentials deviate significantly from the expected distributions and in particular, compact conformations are more underrepresented than open ones (Fig. S6). Error bars indicate that deviations from expected distributions of contacts, are more consistent across potentials type I, V and VI. This is not the case of potentials type II, III and IV (Fig. S6).

Potentials type I favor structures with less number of contacts (i.e. open conformations), and types II deviate toward compact conformations. Figure S7 shows examples of the most and least common structures per type of potential. Notice the reduced number of contacts in potentials type I, even for the most common conformation (Fig. S7A). As shown before, repulsive heteromonomeric interactions (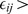
0) promote *c* (Fig. 3 and 5). In the case of an additional repulsive homomonomeric interaction (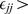
0 in potentials type I), the distribution of conformations shifts considerably towards open conformations (Fig. S6, I & II). A similar effect is observed by comparing potentials type III, IV and V. The addition of repulsive interactions in potentials type IV and V, have a slight impact on the unfavored open conformations observed in potentials type III (Fig. S6, III).

In summary, the potential energy function affects the monomer composition of non-degenerate sequences and the compactness of conformations. The symmetry of the potential, defined as the proportion of attractive and repulsive forces in homo versus heteromonomeric interactions, favors the unbiased use of genotype space and viceversa. Moreover, the relative increase of repulsive over attractive interactions, favors open conformations.

### The designability of phenotypes

In the previous sections I observed that first, potentials vary in their propensity to induce the folding of sequences and structures. Second, potentials favor the viability of regions of sequence and structure space with biased sequence composition and compactness. Here I turn more closely to the relation between sequence and structure across maps. In particular, the relation between the number of sequences per structure, or designability (see *Models*).

Designability (C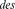) is known to distribute heterogeneously over conformations [16], and this property of a phenotype, has important implications for protein evolution and design. Designable structures, those that map to many sequences, are more likely to be found by a random search across genotype space and are, by definition, more resistant to mutations.

**Figure 7:**
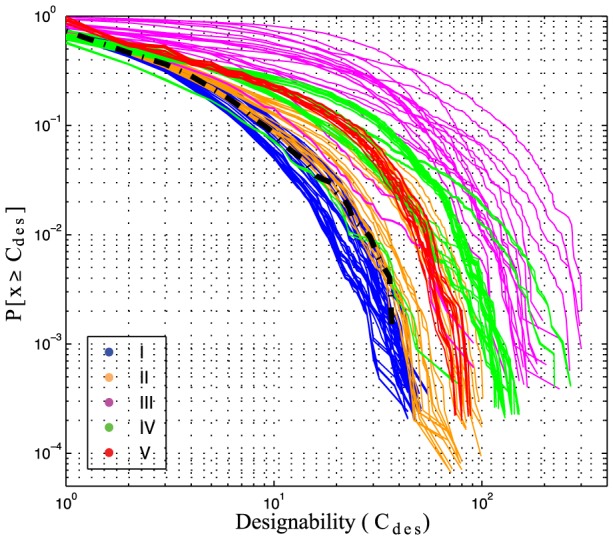
Cumulative probability distributions of the designability of neutral sets for potential types I-VI. For each sequence-structure map I calculate the probability of finding, among non-degenerate sequences, a genotype that folds onto a phenotype with designability C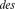 or larger. Here, designability is defined as the number of sequences per neutral set: C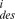 = 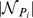 (see *Models*). Color codes according Fig. 3 and Table 1. Dashed black line, HP potential.

In order to study C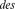 across the phenotype space of a sequence-structure map, I calculate the probability of finding, among non-degenerate sequences, a genotype that folds onto a phenotype with designability C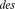 or larger. Figure 7 shows such probabilities as logarithmic cumulative distributions for different types of potentials. As studied before, in the case of the HP model, the probability of finding a phenotype with 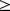
C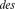, distributes approximately exponential in the 2D lattice [16], [50]. I confirm this trend for potentials type I and II. Other potentials, however, deviate strongly from an exponential distribution.

In the case of potentials type I and II, the probability of finding sequences that map to increasingly designable phenotypes decreases fast compared to the rest of the potentials and is similar to the HP model (black dashed line, Fig. 7). The opposite is true for potentials type III and VI. For instance, in the case of potentials type I, the probability of finding a non-degenerate sequence that maps to a phenotype with C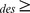 10, is approximately 0.05; while in the case of potentials type III, with the same probability, one finds maps with C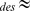 110 sequences. Potentials type V, on the other hand, distribute narrowly and with probability 0.05, presents neutral sets of at least 40 sequences.

Different degrees of variation across potentials of the same type (e.g. contrast potentials type V and III), are the result of the differential distribution of 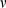 and 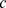. For instance, potentials type III, that show increasing 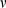, while keeping relatively constant values of 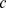, present the broadest C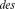 distributions. In contrast, potentials type I, with 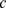 increasing almost linearly with 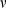, the probability of finding larger C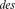, decreases rapidly. In contrast, potentials type V present conserved values of 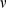 and 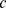, which translates on narrower probability distributions (confront Fig. 5 and 7).

As suggested by a previous study, the identity of designable phenotypes is largely influenced by the potential [50]. As noted above, there is considerable overlap among the phenotypes induced by the potentials studied here. In order to explore this observation further, I group potentials according to their type, rank phenotypes by designability and consider the top and bottom 1 percentiles. There are only 122 conformations (2% of the average number of conformations per potential) encoded by every potential. There are no universally designable phenotype across the potentials studied in this work. I observe that with the exception of potentials type V, the most and least designable phenotypes are unique to each type of potential. Figure S7 shows examples of these phenotypes.

Recall that genotypes of the same neutral set (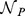) are not necessarily connected (see *Models*). Therefore, from an evolutionary standpoint, instead of 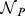 and C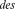, one should rather look at the size of neutral networks (C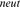). The reason is that the connectivity of genotypes that are part of the same 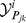, allows them to mutate while preserving the same phenotype (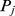). Here, the super and subscripts, stand for the neutral network 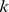 of phenotype 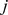, in genotype component 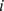 (see *Models*). Figure S8 shows the cumulative probability distribution of 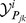 size, across maps. As expected, the probability of finding 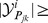
C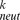, decays faster compared to C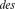 of neutral sets. This trend is particularly clear for potentials type II, III and V. In the case of potentials type III, for instance, the probability of finding neutral sets with 10 or more sequences, reaches values of 0.8; whereas finding neutral networks of similar size, only occurs at probabilities of 0.25. The trend is also evident for potentials type II and V. For instance, with probability of 0.05, one finds neutral sets of 20 and 40 sequences, respectively; whereas, with the same probability, one finds on average neutral networks of only 6 and 8 sequences, respectively. In contrast, the probability distributions of neutral networks and sets, are very similar in the case of potentials type I (see below). In both cases, with probability of 0.07, one finds cluster of sequences of approximately 10 sequences or larger.

These observations suggest that, in addition to variation on the available fraction of sequences and conformations (*i.e.* 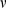 and 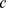), there are considerable differences in C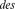 and C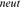 across potentials. Although different types of potentials induce similar sets of phenotypes, the identity of the most and least common phenotypes vary considerably. Additionally, potentials induce differential allocation of sequences across connected components (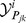), which suggests influences on the size and distribution of neutral sets and neutral networks across genotype space. In the next section, I explore this aspect in more detail.

### Networks of sequences and connected components in genotype space

As described in *Models*, non-degenerate sequences in genotype space can be construed as graphs. In order to investigate the impact of the potential on the distribution of sequences in genotype space from a network perspective, I look at the expected size of connected components (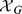), neutral sets (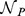) and neutral networks (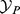) across different maps. I calculate the *expected size* of a cluster of sequences (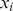) from a collection of sets, **x**, as: 
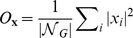
; where 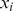 are particular instantiations in the set **x**: 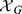, 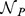 or 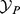. 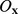 simply computes the weighted average of sequences by their corresponding component size. Because every sequence is multiplied by its component's size; 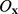 is equivalent to sum over the squares of the size of each component. If we were to choose a random non-degenerate sequence, from genotype space; 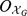 would represent the expected size of the genotype component to which 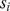 belongs; 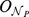, the expected *designability* of its phenotype and 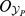, the expected *neutrality* of the neutral network associated to the same phenotype.

**Figure 8:**
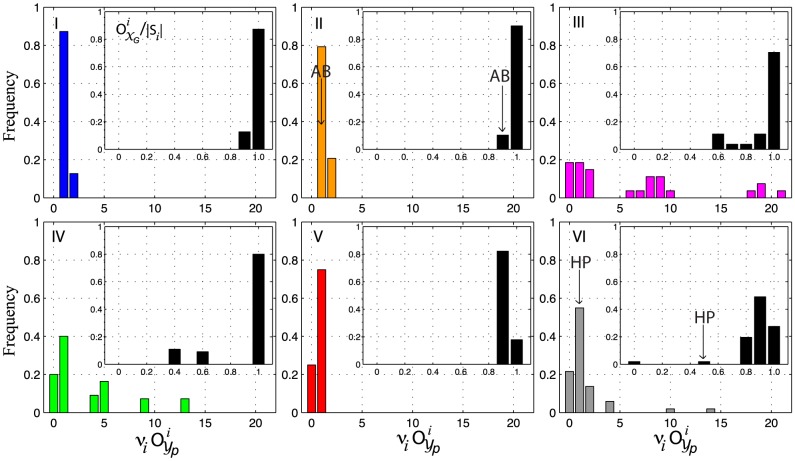
Distribution of expected size of clusters of sequences in genotype space for potentials type I-VI. For each sequence-structure map 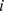, generated by potential 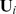, plots present the expected size of sequence clusters 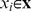 (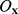), where **x** is: 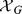 or 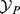 (see main text). Panels present the relative distribution of 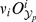 for potentials type I-VI. Distributions are normalized by 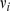, the non-degeneracy of sequence-structure map 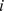. Color code according to Fig. 3 and Table 1. Insets, relative distribution of expected size of genotype components (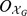), normalized by the total number of non-degenerate sequences (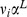 = 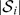).

Figure 8 shows the distributions of 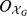 and 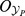 per type of potential. In order to compare maps generated by different potentials, I scale expected size by non-degeneracy (see legend of Fig. 8). Potentials type I, II and V, show genotype components that span on average 97, 99 and 93% of non-degenerate sequences, respectively (insets Fig. 8I, II, V). Note, however, that these distributions of expected size are generally due to the presence of a large genotype component. Figure S9 shows the distribution of the diameter (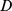) of genotype components per type of potential (see *Models*). While 60 to 90% of genotype components of potentials are composed of a single sequence (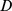 = 0), all types of potentials show at least one large spanning genotype component (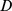 = 18) (Fig. S9).

In addition, potentials type I, II and V, as confirmed by designability of neutral networks in the previous section (Fig. S8), present small neutral networks mostly composed of 2 sequences (Fig. 8). Figure S10 shows the distribution of neutral networks diameter across potentials. Potentials type I and V do not show neutral networks with 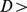
9. Maximum diameter observed for potentials type II is 11.

In contrast to potentials type I, II and V; III, IV and VI, present genotype components and neutral networks that deviate towards smaller and larger expected size, respectively (Fig. 8, S9, S10). Although giant components dominate in the case of potentials type III and IV (Fig 8), they also show cases where genotype components' expected sizes reduce to 60 and 40% of non-degenerate sequences, respectively. In both cases the expected size of neutral networks increases up to 120 and 60 sequences, respectively (without scaling by 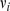). Potentials type IV and VI present neutral networks of diameters up to 14 and potentials type III show cases of neutral networks that cross genotype space (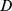 = 18).

Random graph theory predicts that the diameter of a neutral network (D(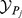)) is a function of the *average neutrality* of sequences that compose the network (see *Models*). The theory predicts the existence of a critical value 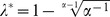 [65]. If the average neutrality of sequences in a network of phenotype 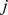 (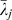), is larger than the critical value (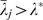), then, sequence in 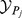, percolate across genotype space and form a giant component. For a binary alphabet, 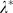 = 0.5.

Overall, potentials sampled in this work show low 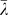 (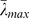 = 0.33) (Figure S11); and, the maximum diameter of neutral networks for potentials type I, II, IV, V and VI; are 9, 11, 14, 9, and 14, respectively (Fig. S10). Five potentials type III, however, present at least one neutral network with D = 18. Moreover, the average neutrality of these neutral networks is 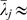
0.15–0.19. There are at least 2 reasons for this disagreement with the theory. First, random graphs may not be a good approximation for neutral networks in the L18 model and/or potentials type III. Results presented in a previous section (*i.e. the use of sequence and structure space*), support this hypothesis. Second, it might be the result of *finite size effects* in the L18 model. In order to test the second hypothesis, 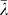 should be calculated at the asymptotic limit [65], an analysis that is beyond the scope of this work.

### Relative distribution of genotype components and networks

As explained in *Models*, sets of sequences that fold onto the same conformation (i.e. neutral set) can be composed of more than one neutral network (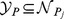). Similarly, genotype components can be composed of more than one neutral set (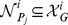) (Fig. 1).

**Figure 9:**
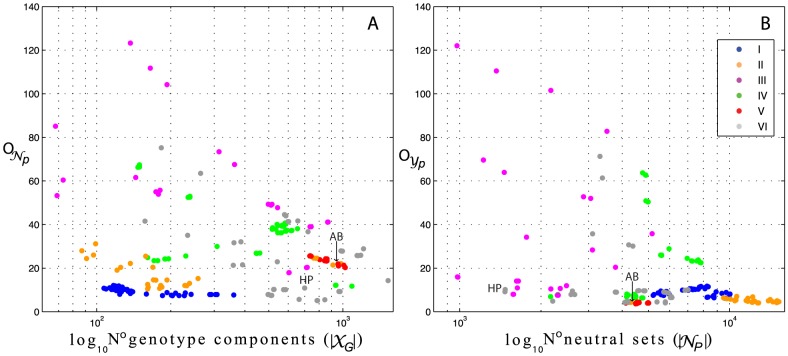
Number of genotype components and neutral sets versus the expected size of neutral sets and neutral networks. (A) Number of genotype components versus the expected size of neutral sets. (B) Number of neutral sets versus the expected size of neutral networks. Expected size of 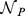 (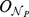) and 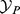 (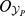), are calculated as the weighted average of sequences across neutral sets and neutral networks, respectively (see main text). Color code according to Fig. 3 and Table 1

In order to explore these differences on the architecture of sequence-structure maps from a broader perspective, I look at 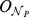 and 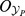 as a function of the number of genotype components (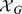) and number of neutral sets (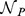), respectively (Figure 9). Each point in Figure 9 is a sequence-structure map induced by a potential of a given type (color code, Fig. 3, Table 1).

The number of 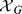 and 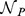, vary approximately one order of magnitude across different potentials. However, compared to 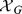, there are ten times more 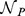 (Fig. 9). As the number of 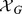 increases, the space gets partitioned into more components and the expected designability of phenotypes (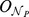) decreases proportionately (Fig. 9A). Potentials type I show few number of components (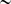
100–400) that contain on average, a large number of neutral sets (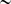
5,000–10,000 - Fig. 9B), of small expected size (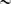
10 sequences - Fig. 9A). Similarly, potentials type II (and V), induce maps with fewer (and larger) 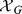, with relatively larger (and smaller) 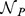, respectively (Fig 9B). Potentials type I, II and V show, on average, small 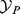 (*i.e.* low neutralities).

In contrast, potentials type III and IV show genotypes components of vastly different sizes. These potentials are enriched on sequences of the same phenotype and consequently, their maps show low encodabilities (x-axis, Fig. 9B). Strikingly, the expected designability of some potentials type III, decreases almost linearly as function of the decimal logarithm of 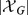, approximately as 15% per order of magnitude (Fig. 9A). The number of 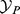 decreases rapidly once encodability reaches values of 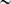
5,000 phenotypes (Fig. 9B).

**Figure 10:**
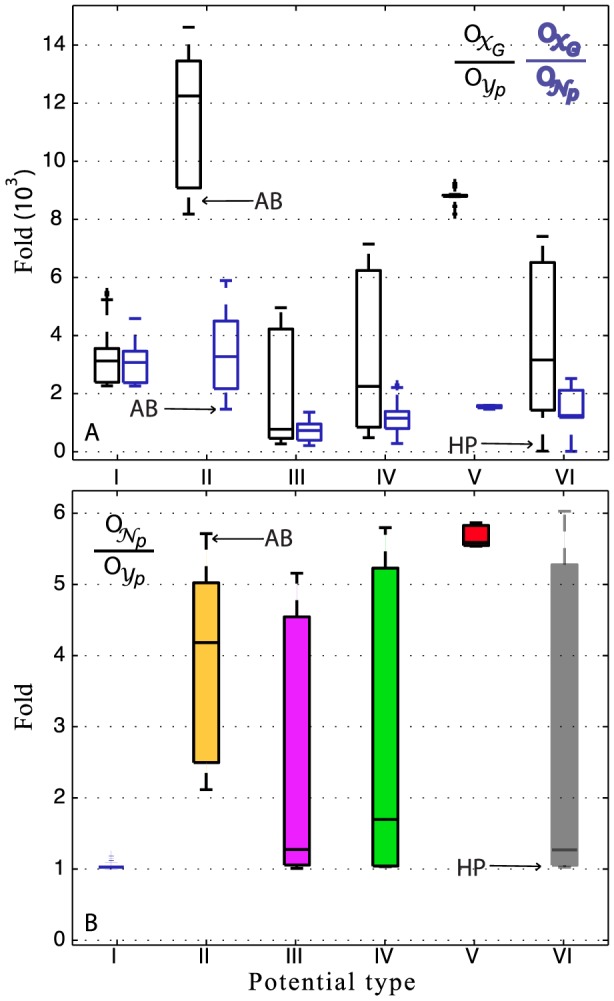
Ratio between expected size of sequence clusters for different types of potentials. (A) Ratio between the expected size of genotype components (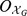) and neutral networks (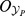) in black. Ratio between the expected size of genotype components (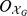) and neutral sets (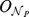) in blue. (B) Ratio between the expected size of neutral sets (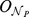) and the expected size of neutral networks (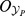). Color code as in Fig. 3 and Table 1. The expected size of a cluster of sequence is calculated as the weighted average of sequences per cluster size (see main text).

The ratio of the expected size of different sequence clusters shows that genotype components are approximately 1,000 to 3,000 fold larger than the expected size of an average neutral set across potentials (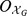/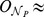
1,000–3,000) (Figure 10A). Although in general the expected size of an average neutral network follows a similar proportion, potentials type II and V, show large deviations, with genotype components: 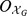/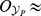 9,000 to 12,000 fold larger than the expected size of neutral networks. These ratios are particularly well conserved across potentials type V (Fig. 10A).

A similar analysis comparing the expected sizes between neutral sets and neutral networks, shows major differences across potentials (Fig. 10B). Strikingly, and as anticipated (see section *the designability and neutrality of phenotypes*), potentials type I show exclusively fully connected neutral sets (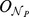/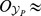 1). In contrast, potentials type V present neutral sets on average 5 to 6 times consistently larger than the expected neutral network. With the exception of potentials type II, that shows on average 4 to 5 neutral networks per phenotype (Fig. 10B); the rest of the potentials show large variation with predominantly 1 to 2 neutral networks per phenotype.

In summary, potentials type I, II and V, induce sequence-structure maps of relatively similar organizations. These potentials show large genotype components and on average few sequences per phenotype. With the exception of potentials type V, however, I and II show on average few genotype components.

Potentials type I show neutral sets composed of a single neutral network and on average, 3,000 networks per genotype components. As seen before, these networks possess short diameters. Similar to potentials type I, type II show approximately 3,000 neutral sets per genotype component. These types of potentials, however, show on average, neutral sets 4 times larger than the expected size of a neutral network. Potentials type V, on the other hand, show on average, 1,800 neutral sets per genotype component and these neutral sets are consistently composed of 5.5 to 6 times more neutral networks.

In contrast, potentials type III, IV and VI, induce sequence structure maps with genotype components and neutral sets of a wide variety of sizes. These potentials show long-tailed distributions of neutral networks per phenotypes, with on average 1 to 2 networks per neutral set. In addition, they show approximately 1,000 to 2,000 neutral sets per genotypes component. While potentials IV and VI reach neutral sets and networks of expected size up to 70 sequences, potentials type III shows neutral networks of up to 120 sequences.

### The phenotypic diversity of genotype neighborhoods

Although the distribution of sequences in genotype space, and in particular of neutral networks, informs on the abundance of phenotypes and their expected mutational robustness, it does not tell us about the mutational divergence between different phenotypes. The differential accessibility to phenotype variants across genotype space has a profound impact on the ability of sequences to produce new, unobserved phenotypes.

In order to study the relative accessibility of sequences to new phenotypes, I consider the phenotypic diversity of a pair of 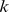-neighborhoods centered at 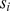 and 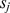 (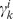, 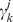) (see *Models*). I calculate the overall fraction of phenotypes *unique* to each of the two *k*-neighborhoods at distance 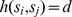, and constant 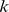, as: 
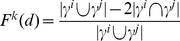
. 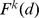 measures the overall diversity of two phenotype neighborhoods as a function of their divergence in genotype space. Note that non-overlapping *k*-neighborhoods only occur at 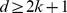 [8], [66].

**Figure 11:**
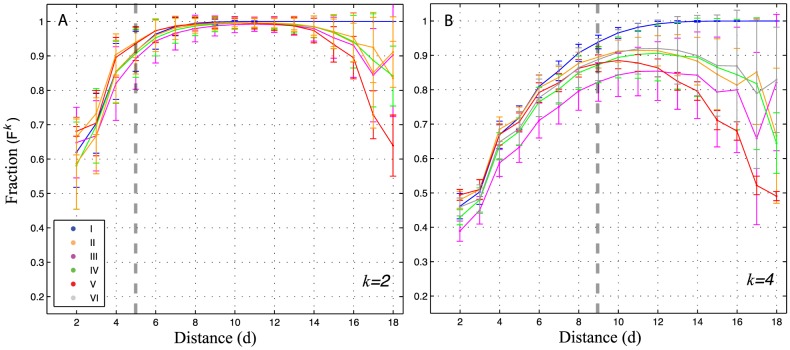
Fraction of new phenotypes across *k*-neighborhoods at distance *d*. For each of the 245 potentials analysed in this study (Table S1 in Text S1), I draw 1,000 random non-degenerate sequences and for each pair of sequences (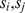), calculate 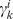, 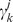 and 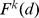, at constant *k* and variable distances *d*. I average 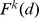 values according to their type of potential (I-VI) (color code, see Fig. 3 and Table 1). (A) *k* = 2. (B) *k* = 4. Error bars represent one standard deviation from the mean. Grey dashed lines illustrate the overlapping threshold: 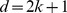.

Figure 11 presents 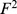 and 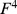 as a function of distance for potentials type I-VI. At very short distances (with even overlapped neighborhoods), 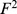 shows 50 to 70% of unique phenotypes (Fig 11A). As expected, at short 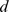 and larger 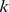, 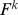 decreases as a function of 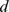 (Fig. 11B). In the case of 2-neighborhoods, the fraction of unique phenotypes increases rapidly with distance and, at short 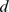, there are only slight differences between types of potentials. At the overlapping threshold of 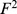 (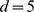, dashed line Fig. 11), approximately 85 to 95% of phenotypes are unique to pairs of neighborhoods. At larger distances, however, 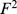 differ considerably across potentials. For instance, at 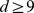, potentials type I access 2-neighborhoods with 100% new phenotypes; whereas, distant 2-neighborhoods of potentials types II, III, IV and VI, share from 10 to 15% phenotypes. This trend intensifies in the case of potentials type V, that reach similar 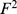 values compared to 2-neighborhoods at short distances (Figure 11A).

In the case of larger 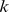-neighborhoods, differences between potentials discussed above become more evident (Fig. 11B and S12). Strikingly, potentials type I consistently find unique phenotypes at 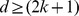. In stark contrast, potentials type V, recover completely the levels of 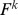 observed at short distances in a fairly symmetric pattern (Fig. 11B, S12).

In order to further explore these differences I look at maximal distances (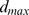) between sequences that are part of the same neutral set, that is, sequences that fold onto the same phenotype. Figure S13 shows such distributions per type of potential. As expected, potentials type I show short maximal distances, with 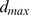 hardly larger than 7 point mutations. In contrast, all other potentials show phenotypes at varying distances and sequences at opposite sides of genotype space (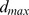 = 18). In particular, potentials type II, IV and VI show 40 to 60% of phenotypes with 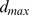 = 18. Consistent with the patterns observed in Fig. 11 (and S12), potentials type III and V show on average, 70 and 97% of phenotypes with 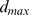 = 18, respectively (Fig. S13).

The existence of sequences at 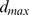 can be explained by the degree of symmetry between attractive and repulsive interactions in the potential. A sequence folds onto its native conformation by stabilizing a set of contacts (2 to 10 in the case of the L18 model). In the case of a completely symmetric potential (as type V), sequences in opposite sides of genotype space, those with every 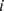 position mutated by the opposite monomer, would preserve the same fraction and type of interactions, and therefore stabilize the same phenotype. In contrast, an asymmetric potential, as potentials of type I, with a single homomonomeric attractive interaction, will populate phenotypes at biased compositional classes. As these observations predict, potentials type III, a fully symmetric potential with no competing interactions, stabilizes sequences at a varying range of distances (Fig. S13).

In summary, potentials induce maps with variable degrees of phenotypic diversity and divergence between neighborhoods. At short mutational distances, there is a large fraction of phenotypic diversity and these values are consistent across types of potentials. At moderate and long distances, however, potentials differ extensively in the distribution of unique phenotypes and those differences are due to the symmetric distribution of the potential's attractive and repulsive forces in homo versus heteromonomeric energy terms.

### Foldability

Not all non-degenerate sequences are guaranteed to fold readily onto their native conformations. The propensity of a polypeptide to fold fast is an important determinant of how protein-like is a random sequence [67]. Next, I look at the impact of the potential on *foldability* (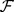), a measure of a sequence's propensity to fold (see *Models*).

Foldability is very sensitive to parameters in the potential. As shown in Figure 4, 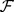 varies extensively across sequence-structure maps, even among those induced by potentials of the same type (see Fig. S14). Min and max median values are −8.2 and −2.3, respectively (the lowest the foldability, the faster the folder - see Eq. 2). Similar values of foldability are also observed to correlate very well with the accessible set of genotypes (Figure S1). The canonical potential HP has a notorious long-tailed distribution biased towards fast folders. This is however not a peculiarity of the HP model, and similar protein-like sequence-structure maps are observed in the case of potentials type I, II and other potentials type VI (Fig. 4 and S1). In addition, variation on the foldability of maps induced by the same potential type, suggests that foldability is highly sensitive to changes on the potential (confront for instance, potentials type I or II in Fig. 4 and S14).

**Figure 12:**
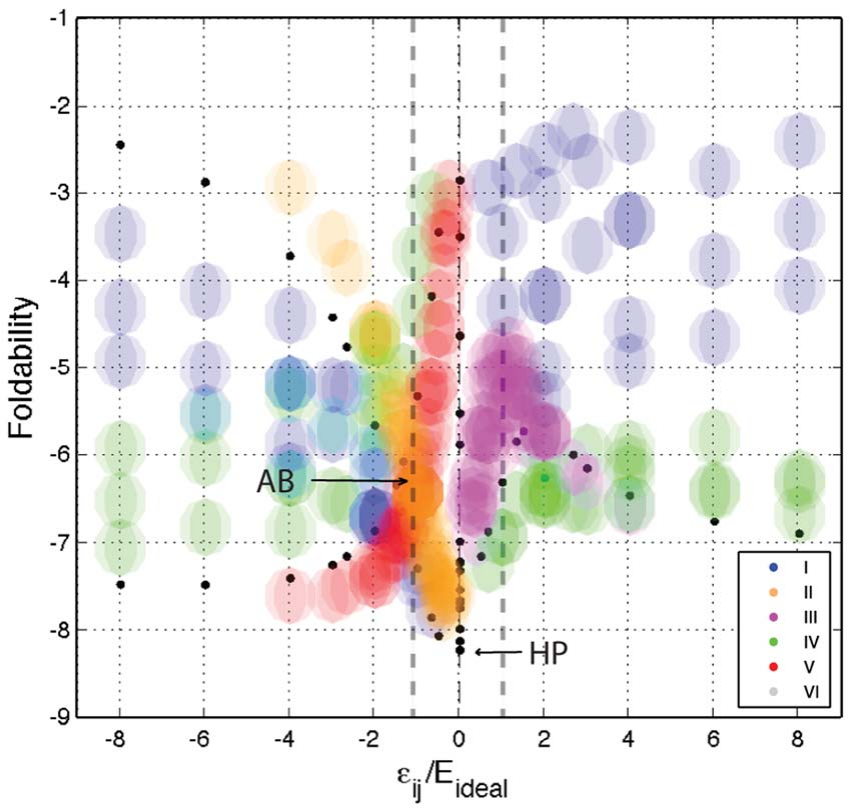
Foldability as a function of a potential's additivity. Foldability was calculated using Eq. 2. Additivity, as described in *Models*. Values refer to the median foldability across non-degenerate sequences for a given potential. Shaded circles correspond to a single potential colored as defined in the legend (see Fig. 3 and Table 1). Black dots represent potentials type VI. Dashed lines illustrate additive potentials (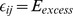; 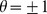).

Evidence from the theory of protein folding relates foldability to cooperativity or the non-additivity of interactions [15]. In the context of binary potentials, I measure additivity (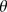) as deviations of *excess* from the *ideal* part of the potential (see *Models*). Figure 12 presents the median 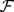 across all non-degenerate sequences of each sequence-structure map, as a function of 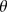. In the case of a completely additive potential: 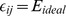 (dashed lined at 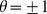).

The association between 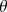 and 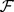 for potentials type I-V is delineated by the foldabilities of potentials type VI (black dots in Fig. 12). This is due to the fact that transitions between types of potentials occur whenever 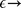 0.0 (grey planes in Fig. 3). Sequence-structure maps that favor foldability are induced by HP-like energy functions, which include potentials type I, II and VI.

Not every potential that deviates significantly from additivity ensures a foldable map. Figure 12 shows that the extent to which additivity dictates the overall 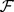 of a map, is a function of the type of interactions present in the potential. For instance, potentials type II and III reach better 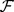 as 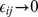; whereas in the case of potentials type V, when 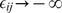.

In summary, our observations confirm the impact of a potential's non-additive interactions on favoring protein-like sequences. I observe that the role of non-additivity is highly dependent on the form of the potential and that different potentials can induce maps as protein-like as the canonical HP model. HP-like sequence-structure maps are particularly induced by potentials type I and II. Most notably, this analysis suggests that by controlling for the form of the potential, it is possible to design a map with a desired fraction of protein-like sequences.

### The binary potential energy functions of natural amino acids

How random are the pairwise interactions observed in the natural amino acid alphabet? I assess this question by comparing the pairwise interactions of amino acids in the Miyazawa-Jerningan (MJ) potential (Table VI in [29]), to the unbiased random sample of potentials studied in previous sections.

I start by counting all pairs of natural amino acids in the MJ potential (*i.e.* 190), and classify them according to the definition in Fig.3. The MJ potential presents all 7 types of potentials analyzed in this work. Because of its continuous energy values, there are only six binary potentials with neutral interactions (*i.e.* type VI), and for convenience, I neglect them in this analysis.

**Figure 13:**
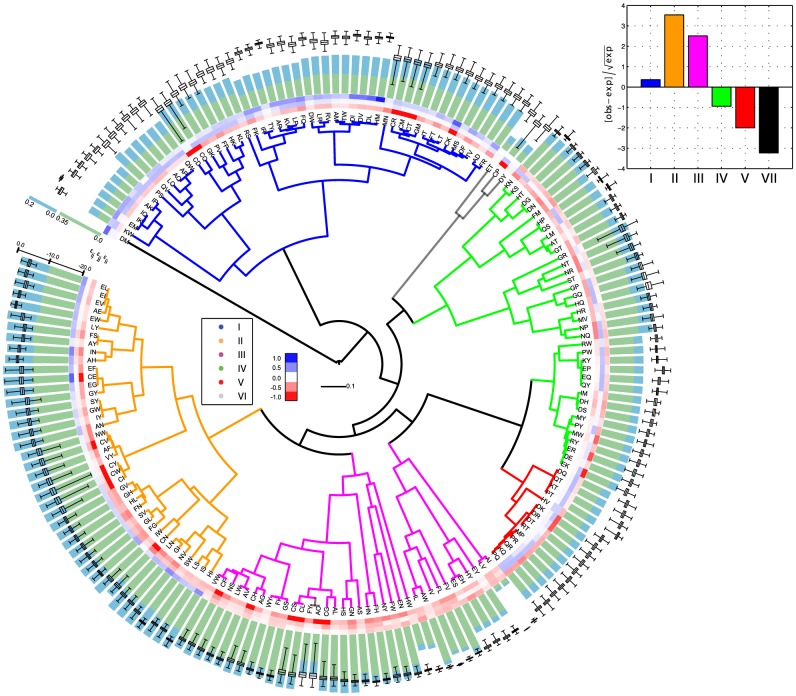
Hierarchical clustering of phenotype spaces generated by binary sequence-structure maps of a statistical potential derived from natural proteins. Values of the pairwise potentials for natural amino acids were obtained from the Miyazawa-Jernigan potential, Table VI [29]. (See legend of Figure 4 and main text).

According to Fig. 3, an homogeneous sample from the space of binary potentials produces potentials type I:II:III:IV:V:VII in the ratio 2∶1∶1∶2∶1∶1. The histogram in Fig. 13, shows a comparison between expected versus observed types of binary potentials in the MJ energy function.

This analysis shows that natural amino acids tend to avoid purely repulsive potentials (type VII) as much as they promote HP-like potentials (type I and II). Strikingly, there is a strong overrepresentation of potentials type III, approximately equivalent to the overall underrepresentation of potentials type IV and V.

In order to gain further insights on the properties of binary combinations of natural amino acids, I perform a similar analysis as the one reported in Figure 4 (see Figure S15).

In contrast to Fig. 4, the clustering of sequence-structure maps of binary potentials of natural amino acids seems more homogeneous. Similar to our previous observations, non-degeneracy ranges from 2 to 34% and encodability, from 1 to 21%. Several sequence structure maps reflect good foldabilities. These maps usually involve strong interactions such as Cys. Sequence-structure maps with different degrees of protein-likeness are observed in the case of potentials type I, II, IV and VI.

The purpose of this analysis is not to argue that these binary potentials reflect the architecture of the sequence-structure map of natural proteins; but to suggest that, if the combination of potentials can be approximately considered additive, then combinations of these binary interactions may indeed reflect some of the properties of sequence-structure maps induced by larger alphabets. Indeed, random libraries composed primarily of 3 amino acids, such as AEK [68] and QLR [57], can be decomposed into potentials type I, II, IV; and I, I, V; respectively.

In summary, these results show that the types of binary potentials observed in an unbiased sample of the space of energy functions, are represented in the interactions of natural amino acids, as described by the MJ potential. The types of potentials overrepresented in proteinaceous amino acids are potentials characterized as HP-like. Furthermore, these analysis suggest that random libraries enriched in HP-like potentials, are likely to favor protein-like sequence-structure maps.

## Discussion

A graph theoretic approach, inspired on the concept of genotype-phenotype map, provides a common quantitative framework to investigate the sequence-structure relation. According to this framework, viable genotypes are represented as nodes, and edges connect genotypes that differ in a single position along the sequence. The distinction of genotypes according to the phenotypes they map onto, induces subgraphs whose properties and distribution have important consequences for biology. These subgraphs can be characterized quantitatively in terms of the statistics of their expected sizes, diameters and distances. I refer to this detailed characterization of the sequence-structure map, as its architecture.

In this study I showed that the potential affects the architecture of the sequence-structure map and, that its impact on some of the map's properties is highly predictable based on features of the potential.

First, the balance between attractive versus repulsive interactions in the potential, affects the available fraction of sequences and structures, and also induces biases towards compositional classes and the compactness of conformations. Second, although potentials induce similar sets of phenotypes, the identity of the most and least common phenotypes, differs. Third, potentials affect both the number, expected size, and the relative distribution of genotype components, neutral sets and neutral networks. Fourth, the overall symmetry of the potential, defined as the distribution of attractive and repulsive forces in homo versus heteromonomeric interactions, predicts the phenotypic diversity of genotype neighborhoods across divergent regions in sequence space. Fifth, foldability varies considerably across both potentials of different type, and potentials of the same type that preserve similar non-degeneracies and encodabilities. I observed that the predictability of a potential's non-additive interactions on the average foldability of a sequence-structure map, depends on the type of potential. Sixth, binary potentials of natural proteins, as defined by the MJ energy function, present biases that overrepresent HP-like potentials.

In order to interpret these results in the context of the sequence-structure map of real peptides, one should be aware of the limitations of SEMs and the meaning of the energy terms in the potential. In the following, I discuss these limitations and evaluate critically the results presented in this study.

Previous explorations of binary alphabets showed that repulsive interactions reduce the overall degeneracy of sequences, increasing the available fraction of viable genotypes and phenotypes [39]. The results in the present study confirm this observation and by distinguishing between homo and heteromonomeric interactions, show that non-degeneracy is promoted by potentials with predominantly attractive interactions (type II and III, Fig. 3 and Table 1), whereas encodability is only promoted by a combination of attractive homomonomers and repulsive heteromonomers (type II).

A second observation anticipated by SEMs is the effect of repulsive interactions on the compactness of conformations [39]. Repulsive interactions tend to induce conformations with less number of contacts. The results in the present study reveal that not every repulsive interaction induces this effect. Indeed, only a combination of repulsive interactions at both homo and heteromonomeric interactions, reduces compactness (type I). In the case of repulsive homomonomer or heteromonomer interactions only, the effect is either none or opposite, respectively (type V and II).

Previous studies pointed out that the effect of repulsive interactions is due to the avoidance of local energy minima and the distinction between conformations, by the induction of larger energy gaps [39]. The results in the present work confirm this intuition. Potentials with a larger average fraction of repulsive interactions show better foldabilities (see below).

Several studies using different alphabet sizes, potentials, and polymer lengths, suggest that designability arises under a large variety of parameters [50], [69], [70]. Some of these studies, using maximally compact conformations, have shown that designability is affected by the potential and that, although different potentials induce a similar set of phenotypes, the most and least common phenotypes vary considerably across potentials [50]. The results presented here confirm these observations in the L18 model, with a full enumeration of the conformational space; and show that due to the differential induction of non-degenerate sequences and encodable conformations, potentials induce maps with variable degrees of designabilities. Similarly, I showed that the neutrality of networks presents analogous trends compared to the designability calculated over entire neutral sets. I showed that their relation depends on the type of potential.

In the present study I explored three additional properties of sequence-structure maps, and their dependence on the potential energy function. Firstly, by considering the expected size of genotype components, neutral sets and neutral networks; I observed that potentials induce a large variation on the relative distribution of sequences and structures in genotype space. Strikingly, there are significant differences on the number of neutral networks per phenotype and the fraction of networks per genotype component across potentials.

Secondly, as a consequence of different non-degeneracies and encodabilities observed across maps, as well as the variation of expected size of neutral sets and neutral networks, sequence-structure maps show considerable differences on the phenotypic diversity at divergent distances on genotype space.

Thirdly, I used previous definitions of foldability, based on the energy gap, as a proxy to estimate the extent of protein-likeness across non-degenerate sequences. I observed that not every potential is equally likely to induce good folders. Most notably, non-additive potentials induce lower values of foldability. However, this prediction depends on the type of potential. Among these, are potentials that also show optimized levels of non-degeneracy and encodability (*i.e.* type I and II).

Altogether, these results support previous observations on the distribution of sequences across genotype space based on the HP model [42]. HP-like potentials (*i.e.* type I and II), show on average small neutral networks that hardly reach diameters larger than 50% of genotype space. However, in contrast to the HP model, HP-like potentials are not always isolated in genotype space, but part of genotype components of large expected sizes. In part, this is due to the symmetry of the potential, that is, the proportion of attractive and repulsive interactions on homo versus heteromonomeric interactions. In practice, a symmetric potential is one in which interactions can be realized by more than one combination of monomers (*i.e.* redundant). Because the chemistry of the natural amino acid alphabet is known to be redundant, these observations imply that, as long as types of amino acid interactions in the structure are preserved, neutral networks (or at least, neutral sets) are likely to extend over divergent regions of genotype space. Previous, *in silico* analysis, support this observation [71], as do protein design strategies based on conservation patterns of hydrophobic-polar interactions [72].

The results presented here provide a rationale based on the proportion and types of interactions resulting from the monomer composition of sequences. As shown, this rationale makes predictions on the expected phenotypic diversity and the relative distribution of clusters of sequences. In addition, this framework makes further predictions about the distribution of sequences in genotype space and the role of structural determinants of sequence variation. For instance, it predicts the existence of larger neutral networks/sets in the case of structures with high degrees of symmetry. Indeed, studies exploring structural determinants of sequence variation show that designable folds are more symmetric than expected [73], [74]. Moreover, such a framework, suggests a strategy to improve fold assignment, a common task in comparative modeling [75] and in the identification of divergent homologous sequences [76]. This and similar predictions can be tested systematically in the case of proteins with long evolutionary histories, that encompass large superfamilies spanning divergent regions of genotype space (*e.g.* globins [77]; 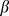-barrels [78]).

In extrapolating these observations to natural polypeptides one should take into account two relevant features of the potential, and evaluate how these features scale with the size of the potential. First, as suggested by previous studies, alphabet size has a fundamental impact on the fraction and diversity of accessible phenotypes [14], [79]. The observations presented here, suggest that a more accurate definition of alphabet size should account for the number and types of *non-equivalent* monomeric interactions. One might consider an *effective* alphabet size as the total number of *chemically non-redundant* pairwise interactions. Such a measure should account for differences between homo versus heteromonomeric interactions, and attractive versus repulsive. This represents a natural distinction between the types of potentials analysed in this work (Fig. 3, Table 1).

In the case of binary potentials, hetero versus homomonomeric interactions are in a 1∶2 ratio. In general, with an alphabet size 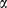, hetero to homomonomeric interactions are in an (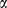-1):2 ratio. Thus, in the case of natural proteins, there are approximately 9 hetero per each homomonomeric interaction. In addition, some of the types of potentials studied here, are more diverse in terms of attractive versus repulsive forces. Overall, because of the diversity of energy values, the alphabet of HP-like potentials must present indeed, large *effective* sizes.

A second important aspect is to what extent, potentials composed of 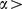
2, can be considered simply as the additive contribution of independent binary potentials. Observations from simulation and empirical results, suggest that some of the properties presented above, for independent pairwise potentials, may apply to sequences composed of larger alphabets.

Firstly, successful energy functions used to distinguish between native and non-native conformations, are based on the additive contribution of pairwise interactions [80].

Secondly, *in silico* mutational studies, show that changes in stability across different types of folds, are normally distributed [81]. This observation implies that most perturbations to the stability of protein structures, are additive.

Thirdly, natural amino acids, as analyzed according to the MJ potential, overrepresent binary energy functions with HP-like features, as do natural sequences (unpublished data). A three-monomer alphabet may involve up to 3 different types of potentials; and a 4- and 5-monomer alphabet could, in principle, encompass up to 6 and 10 different types of potentials, respectively. Considering this observation, it is tempting to suggest an explanation as to why random libraries of polypeptides and protein folds designed using small alphabets, favor some types of potentials. For instance, libraries composed of mainly 3 amino acids such as AEK [68] and QLR [57], present I, II, IV and I, I, V potentials; respectively. Similarly, random libraries constructed of 5 amino acids, such as VADEG, composed of potential types: I, II, III, IV; in a 1∶2∶1∶1 ratio; show high levels of solubility and evidence of secondary structure formation [82]. A related empirical observation comes from the synthesis of protein folds using reduced alphabets. Riddle et al. [83], synthesized the SH3 fold using an alphabet of size 5: AIGEK. This alphabet includes potential types: I, II, III, IV; in a 3∶4∶2∶1 ratio, respectively.

Fourthly, its has been recognized that non-native interactions play an important role during folding [84]. This suggests that although dominant, HP-like interactions would not be the only force required for successful folding, and would explain the relative lower representation of other types of potentials in reduced alphabets and in natural proteins. Other types of protein sequences may serve to test this hypothesis. Indeed, disordered proteins are known to be enriched in interactions that differ considerably to those commonly found in globular proteins [85].

Two sources of bias may appear when comparing the actual natural pairwise potentials to a random sample from the space of energy values.

First, the chemistry of natural amino acids might cause an overrepresentation of pairwise potentials of certain types. Such bias might be explained by either biochemical constraints on the synthesis of a limited amino acid chemistry, or by the influence of natural selection on the amino acids introduced into the genetic code. A second source of bias, due to natural selection, is the differential usage of amino acids in natural proteins. From the proteinaceous amino acid pool, natural sequences might tune their composition and favor types of interactions that promote folding. Since the MJ potential was derived from the propensity of pairwise amino acid interactions in crystal structures of proteins, it might contain a mixture of these biases.

The predominance of some types of potentials in natural proteins, as well as the empirical evidence of random libraries listed above, suggest the existence of constraints on the establishment of a primordial amino acid alphabet. Studies exploring the average solubility of random libraries have demonstrated a strong variation of protein-like features in these libraries, as a function of amino acid composition. Indeed, the so called primordial amino acids, have been shown to promote solubility and the formation of secondary structure [82]. The analysis presented here can be used to fully enumerate potentials that are likely to meet these constraints. Such analysis may provide a quantitative method to test the likelihood of reduced amino acid alphabets.

Conversely, conjectures about the use of larger alphabets suggest the expansion of phenotype space [61]. In a forthcoming publication I explore larger amino acid alphabets, and quantitative ways of evaluating the effect of combinations of different types of potentials on the architecture of the sequence-structure map of natural proteins.

## Supporting Information

Figure S1
**Hierarchical clustering of genotype spaces generated by the sequence-structure maps of binary potentials.** Artificial potentials were constructed considering 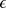 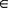 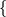
−1.00, −0.75, −0.50, −0.25, 0.00, 0.25, 0.50, 0.75, 1.00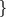 (see main text and Table S1 in Text S1). Canonical potentials are the HP and AB models and their respective shifted versions (see Fig. 1 and main text). Hierarchical clustering was carried out using similarity measure based on genotype space, 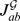, and the group-average method. 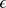 values of each potential are specified on a color scale at the branches' tips, with 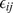 specified by the outermost value. Branches are colored according to the 7 different potentials described in Fig. 3. Green and blue stacked bars following the color-coded potentials, correspond to non-degeneracy and encodability, respectively. Boxplots, in black, represent the distribution of median foldability values over non-degenerate genotypes for each map. Canonical potentials are the HP and AB models and their shifted versions (Fig. 2). They are highlighted with red dots.(EPS)

Figure S2
**Hierarchical clustering of phenotype sets generated by canonical and artificial potentials using different clustering methods.** Top. Single linkage. Bottom. Complete linkage. Hierarchical clustering was carried out using the Jaccard index, 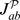, described in the main text. See legend Figure S1.(EPS)

Figure S3
**Non-degeneracy and encodability for the potentials sampled in this study.** Non-degeneracy and encodability represented on the 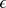 coordinates of Figure 3. (A) Non-degeneracy. The fraction of viable sequences of genotype space. (B) Encodability. Fraction of the conformation space accessible to non-degenerate sequences.(EPS)

Figure S4
**Ideal and excess components versus non-degeneracy and encodability across different potentials.** (A, C). Non-degeneracy and encodability versus a potential's ideal component (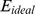). (B, D) Non-degeneracy and encodability versus a potential's excess component (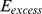). Color represents potential types, as in Fig. 3 and Table 1.(EPS)

Figure S5
**Non-degeneracy and encodability versus the heteromonomeric interaction of binary potenials.** (A) Non-degeneracy (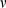) versus 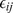. Non-degeneracy stands for the fraction of viable sequences of genotype space. (B) Encodability (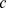) versus 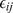. Encodability corresponds to the fraction of the conformation space accessible to non-degenerate sequences. Color represents potential types, as in Fig. 3 and Table 1.(EPS)

Figure S6
**Observed versus expected compactness for potentials types I to VI.** The compactness of a conformation corresponds to its total number of contacts (2 to 10 for the L18 model). I estimate expected compactness for a given map by sampling 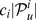 conformations from phenotype space induced by the potential 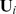. The frequency of each compactness is compared to the observed number of conformations per type of potential. Color represents potential types, as in Fig. 3 and Table 1.(EPS)

Figure S7 **Examples of the most and least common conformations unique to different types of potentials.** For each potential studied here, I rank phenotypes according to their designability and group the 1st highest and lowest percentile according to the type of potential. I select phenotypes that fall into any of these categories and are unique to the particular type of potential. The figure presents the most (A) and least (B) common phenotypes per potential (I-VI). Number of contacts per type of potential: I) 6, 4; II) 10, 9; III) 10, 9; IV) 10, 8; V) 10, 10; VI) 9, 8; for most and least common phenotypes, respectively.
(EPS)

Figure S8
**Cumulative probability distributions of neutrality of neutral networks for potentials type I-VI.** For each sequence-structure map I calculate the probability of finding, among non-degenerate sequences, a genotype that folds onto a phenotype's neutral network with neutrality C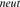 or larger. Neutrality is defined as the number of sequences per neutral network: C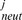 = 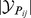 (see *Models*). Color as in Fig. 3 and Table 1. Dashed black line, HP potential.(EPS)

Figure S9
**Distribution of the diameter of genotype components.** For each sequence-structure map and for each genotype component, we compare all-against-all sequences and record the maximum distance observed (*i.e.* diameter, 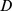). Plots show the average frequency across maps generated by different types of potentials I-VI. Error bars represent one standard deviation from the mean. Color as in Fig. 3 and Table 1.(EPS)

Figure S10
**Distribution of the diameter of neutral networks.** For each sequence-structure map and for each neutral network, we compare all-against-all sequences and record the maximum distance observed (*i.e.* diameter, 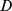). Plots show the average frequency across maps generated by different types of potentials I-VI. Error bars represent one standard deviation from the mean. Color as in Fig. 3 and Table 1.(EPS)

Figure S11 **Average sequence's neutrality per neutral network versus network diameter.** For each sequence of each neutral network of each potential, I calculate its average sequence's neutrality (*i.e.* fraction of sequences in the 1-neighborhood that remains in the network) (see *Models*). Color as in Fig. 3 and Table 1.
(EPS)

Figure S12
**Fraction of novel phenotypes across** ***k*** **-neighborhoods at distance** ***d*** **.** For each of the 245 potentials analyzed in this study (Table S1 in Text S1), I draw 1,000 random non-degenerate sequences and for each pair of sequences (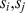), calculate 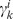, 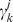 and 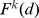, at constant *k* and variable distances *d*. I average 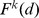 values according to their type of potential (I-VI) (color code, see Fig. 3 and Table 1). (A) *k* = 3. (B) *k* = 5. Error bars represent one standard deviation from the mean. Grey dashed lines illustrate the overlapping threshold: 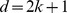.(EPS)

Figure S13
**Distribution of the diameter of neutral sets.** For each map, and for each neutral set, every pair of sequences are compared and the maximum observed hamming distance is recorded (*i.e.* diameter, 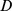). Plots show average frequency across maps generated by different types of potentials I-VI. Error bars represent one standard deviation from the mean. Color as in Fig. 3 and Table 1.(EPS)

Figure S14
**Foldability for the potentials sampled in this study represented on the** 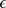 **coordinates.** Every point corresponds to a sequence-structure map. Color represents median foldability calculated across non-degenerate sequences, using Eq. 2. Small dots represent the median foldability calculated over binary potentials of natural amino acids. Binary potentials of natural amino acids are obtained from the MJ potential (Table VI in 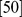).(EPS)

Figure S15
**Hierarchical clustering of phenotype sets generated by natural potentials using different methods of clustering.** Top. Single linkage. Bottom. Complete linkage. Hierarchical clustering was carried out using the Jaccard index 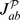, described in the main text. See Figure S1 legend.(EPS)

Text S1 **Supplementary methods and supporting Tables S1 and S2.**
(PDF)
