# Supplementary material for: The Amino Acid Alphabet and the Architecture of the Protein Sequence-Structure Map. I. Binary Alphabets
Source: PLoS Comput Biol. 2014 Dec 4;10(12):e1003946. doi: 10.1371/journal.pcbi.1003946 (PMC4256021; doi:10.1371/journal.pcbi.1003946)
Supplement: Text S1 — Supplementary methods and supporting Tables S1 and S2. (PDF) [file pcbi.1003946.s016.pdf]

Supplementary Information

The Amino Acid Alphabet and the Architecture of the Protein Sequence-Structure Map.

I. Binary alphabets.

Evandro Ferrada

1 Supplementary Methods

1.1 The enumeration and statistics of protein lattice conformations for the L18 model

Exhaustive enumeration of conformations in the two-dimensional L18 simple exact model, was carried out using the *self-avoiding walk* algorithm (SAW) [1, 2]. Each conformation can be encoded by a set of moves that describe the position of each monomer respect to the previous one, along the polymer chain.

Similarly, each conformation can be characterized by a unique set of contacts between monomers. These contact sets can be represented by a symmetric adjacency matrix, where values of 1 indicate contacts between monomers that are not adjacent along the chain. Different conformations can, however, be described by the same contact set. Because, by definition, a non-degenerate sequence must fold onto a unique conformation, the accessible conformational space must be composed only of contact sets that describe a unique conformation.

There are a total of 5,808,335 possible conformations ($\mathcal{P}$) in the L18 model. These conformations map to 170,670 contact sets. There are only 77,635 contact sets that map to single conformations and therefore are potentially encodable. We refer to this set of conformations as the *encodable set* ($\mathcal{P}_u \subset \mathcal{P}$). The encodable set is obtained by simply counting the number of conformations per contact set and selecting the ones that map to single conformations.

The binary L18 model is composed of 2^{18}= 262,144 sequences. To test if a given sequence s_i is non-degerenate (g=1), we fold s_i onto every conformation ($|\mathcal{P}|$ =170,670 contact sets) and calculate its energy using equation 1 (see main text). s_i is defined as non-degenerate if there is a unique energy minimum (E_{min}), on a single conformation of the encodable set.

For each of the potentials studied in this work all 262,144 sequences are enumerated and tested against every possible contact set, as described above. All the software was written in ANSI C.

1.2 Hierarchical clustering and Jaccard index

In order to explore similarities between the foldable fraction of genotype space or the encodable fraction of conformational space, across different potentials, I use *hierarchical clustering* [3]. Briefly, hierarchical clustering aims to group a set of elements into clusters given two main parameters. First, a *metric* of (dis)similarity between pairs of elements; and second, a *linkage* criterion to decide whether two elements belong to the same cluster.

The set of elements that we aim to cluster correspond to the set of genotypes or phenotypes generated by a pair of binary potentials. As described in the main text, a given potential $\mathbf{U}_i$ induces a set of non-degenerate sequences ($\mathcal{S}_i \subseteq \mathcal{G}$), which fold onto a corresponding accessible set of conformations ($\mathcal{C}_i \subseteq \mathcal{P}_u$). In our calculations, the set $\mathcal{K}$ corresponds to either genotype ($\mathcal{G}$) or phenotype space ($\mathcal{P}$); and k to the subsets ($\mathcal{S}$ or $\mathcal{C}$) induced by a pair of potentials.

The metric we use here is the *Jaccard similarity index* (J) [4]. The J between sets k_a and k_b, is defined as:

$$J_{ab}^{\mathcal{K}} = \frac{|k_a \bigcap k_b|}{|k_a \bigcup k_b|}. \tag{1}$$

with k_a, $k_b \subseteq \mathcal{K}$ and $a \neq b$. $|\bullet|$ symbolizes the cardinality of the set $\bullet$. Where $0 \leq J_{ab}^{\mathcal{K}} \leq 1$. If $k_a = k_b$ then $J_{ab}^{\mathcal{K}}=1$; and $J_{ab}^{\mathcal{K}}=0$ if no elements are shared by k_a and k_b. Thus, $J_{ij}^{\mathcal{G}}$ estimates the similarity between the subsets $\mathcal{S}_i$ and $\mathcal{S}_j$. While $J_{ij}^{\mathcal{P}}$ gives the similarity between $\mathcal{C}_i$ and $\mathcal{C}_j$; between two independent sequence-structure maps (i, j).

Several linkage criteria described in the literature capture slightly different properties of the clustering of sets. Among the most commonly used are *single* linkage, *complete* linkage and *group average.*

Briefly, single linkage, also called *nearest neighbor*, uses the smallest distance between elements of the pair of clusters. In contrast, complete linkage, uses the largest distance. Whereas, group average linkage uses the average distance between all pairs of objects in the two clusters [3].

In this study I explore these three linkage criteria (see main text and Figures 4

and S2), for $J_{ij}^{\mathcal{G}}$ and $J_{ij}^{\mathcal{P}}$ (see Figures 4 and S1), and show that they do not affect the conclusions. I use the routine *linkage* implemented in the software Matlab [5], and the graphic online tool iTol [6].

References

[1] Madras N, Slade G (2012) The self-avoiding walk. Springer.

[2] Irbäck A, Troein C (2002) Enumerating designing sequences in the hp model. Journal of Biological Physics 28: 1–15.

[3] Jain AK, Dubes RC (1988) Algorithms for clustering data. Prentice-Hall, Inc.

[4] Jaccard P (1912) The distribution of the flora in the alpine zone. 1. New phytologist 11: 37–50.

[5] The MathWorks I (2012) MATLAB and Statistics Toolbox Release 2012b. Natick, Massachusetts, United States.

[6] Letunic I, Bork P (2007) Interactive tree of life (itol): an online tool for phylogenetic tree display and annotation. Bioinformatics 23: 127–128.

Table S1. Artificial potentials for binary alphabets					
ID	ϵ_{ii}	ϵ_{jj}	ϵ_{ij}	Type	Canonical
P010	-1.00	-0.75	-1.00	III	
P011	-1.00	-0.75	-0.75	III	
P012	-1.00	-0.75	-0.50	III	
P013	-1.00	-0.75	-0.25	III	
P014	-1.00	-0.75	0.00	VI	
P015	-1.00	-0.75	0.25	II	
P016	-1.00	-0.75	0.50	II	
P017	-1.00	-0.75	0.75	II	
P018	-1.00	-0.75	1.00	II	
P020	-1.00	-0.50	-0.75	III	
P022	-1.00	-0.50	-0.25	III	
P024	-1.00	-0.50	0.25	II	
P026	-1.00	-0.50	0.75	II	
P028	-1.00	-0.25	-1.00	III	
P029	-1.00	-0.25	-0.75	III	
P030	-1.00	-0.25	-0.50	III	
P031	-1.00	-0.25	-0.25	III	
P032	-1.00	-0.25	0.00	VI	
P033	-1.00	-0.25	0.25	II	
P034	-1.00	-0.25	0.50	II	
P035	-1.00	-0.25	0.75	II	
P036	-1.00	-0.25	1.00	II	
P038	-1.00	0.00	-0.75	VI	
P040	-1.00	0.00	-0.25	VI	
P042	-1.00	0.00	0.25	VI	
P044	-1.00	0.00	0.75	VI	
P046	-1.00	0.25	-1.00	IV	
P047	-1.00	0.25	-0.75	IV	
P048	-1.00	0.25	-0.50	IV	
P049	-1.00	0.25	-0.25	IV	
P050	-1.00	0.25	0.00	VI	
P051	-1.00	0.25	0.25	I	
P052	-1.00	0.25	0.50	I	
P053	-1.00	0.25	0.75	I	
P054	-1.00	0.25	1.00	I	
P056	-1.00	0.50	-0.75	IV	
P058	-1.00	0.50	-0.25	IV	
P060	-1.00	0.50	0.25	I	

Table S1. Artificial potentials for binary alphabets					
ID	ϵ_{ii}	ϵ_{jj}	ϵ_{ij}	Type	Canonical
P062	-1.00	0.50	0.75	I	
P064	-1.00	0.75	-1.00	IV	
P065	-1.00	0.75	-0.75	IV	
P066	-1.00	0.75	-0.50	IV	
P067	-1.00	0.75	-0.25	IV	
P068	-1.00	0.75	0.00	VI	
P069	-1.00	0.75	0.25	I	
P070	-1.00	0.75	0.50	I	
P071	-1.00	0.75	0.75	I	
P072	-1.00	0.75	1.00	I	
P074	-1.00	1.00	-0.75	IV	
P076	-1.00	1.00	-0.25	IV	
P078	-1.00	1.00	0.25	I	
P080	-1.00	1.00	0.75	I	
P082	-0.75	-0.75	-1.00	III	
P091	-0.75	-0.50	-1.00	III	
P092	-0.75	-0.50	-0.75	III	
P093	-0.75	-0.50	-0.50	III	
P094	-0.75	-0.50	-0.25	III	
P095	-0.75	-0.50	0.00	VI	
P096	-0.75	-0.50	0.25	II	
P097	-0.75	-0.50	0.50	II	
P098	-0.75	-0.50	0.75	II	
P099	-0.75	-0.50	1.00	II	
P100	-0.75	-0.25	-1.00	III	
P101	-0.75	-0.25	-0.75	III	
P102	-0.75	-0.25	-0.50	III	
P103	-0.75	-0.25	-0.25	III	
P104	-0.75	-0.25	0.00	VI	
P105	-0.75	-0.25	0.25	II	
P106	-0.75	-0.25	0.50	II	
P107	-0.75	-0.25	0.75	II	
P108	-0.75	-0.25	1.00	II	
P109	-0.75	0.00	-1.00	VI	
P111	-0.75	0.00	-0.50	VI	
P112	-0.75	0.00	-0.25	VI	
P114	-0.75	0.00	0.25	VI	
P115	-0.75	0.00	0.50	VI	

Table S1. Artificial potentials for binary alphabets					
ID	ϵ_{ii}	ϵ_{jj}	ϵ_{ij}	Type	Canonical
P117	-0.75	0.00	1.00	VI	
P118	-0.75	0.25	-1.00	IV	
P119	-0.75	0.25	-0.75	IV	
P120	-0.75	0.25	-0.50	IV	
P121	-0.75	0.25	-0.25	IV	
P122	-0.75	0.25	0.00	VI	
P123	-0.75	0.25	0.25	I	
P124	-0.75	0.25	0.50	I	
P125	-0.75	0.25	0.75	I	
P126	-0.75	0.25	1.00	I	
P127	-0.75	0.50	-1.00	IV	
P128	-0.75	0.50	-0.75	IV	
P129	-0.75	0.50	-0.50	IV	
P130	-0.75	0.50	-0.25	IV	
P131	-0.75	0.50	0.00	VI	
P132	-0.75	0.50	0.25	I	
P133	-0.75	0.50	0.50	I	
P134	-0.75	0.50	0.75	I	
P135	-0.75	0.50	1.00	I	
P136	-0.75	0.75	-1.00	IV	
P138	-0.75	0.75	-0.50	IV	
P139	-0.75	0.75	-0.25	IV	
P141	-0.75	0.75	0.25	I	
P142	-0.75	0.75	0.50	I	
P144	-0.75	0.75	1.00	I	
P145	-0.75	1.00	-1.00	IV	
P146	-0.75	1.00	-0.75	IV	
P147	-0.75	1.00	-0.50	IV	
P148	-0.75	1.00	-0.25	IV	
P149	-0.75	1.00	0.00	VI	
P150	-0.75	1.00	0.25	I	
P151	-0.75	1.00	0.50	I	
P152	-0.75	1.00	0.75	I	
P153	-0.75	1.00	1.00	I	
P155	-0.50	-0.50	-0.75	III	
P161	-0.50	-0.50	0.75	II	
P162	-0.50	-0.50	1.00	II	
P163	-0.50	-0.25	-1.00	III	

Table S1. Artificial potentials for binary alphabets					
ID	ϵ_{ii}	ϵ_{jj}	ϵ_{ij}	Type	Canonical
P164	-0.50	-0.25	-0.75	III	
P165	-0.50	-0.25	-0.50	III	
P166	-0.50	-0.25	-0.25	III	
P167	-0.50	-0.25	0.00	VI	
P168	-0.50	-0.25	0.25	II	
P169	-0.50	-0.25	0.50	II	
P170	-0.50	-0.25	0.75	II	
P171	-0.50	-0.25	1.00	II	
P173	-0.50	0.00	-0.75	VI	
P175	-0.50	0.00	-0.25	VI	
P177	-0.50	0.00	0.25	VI	
P179	-0.50	0.00	0.75	VI	
P181	-0.50	0.25	-1.00	IV	
P182	-0.50	0.25	-0.75	IV	
P183	-0.50	0.25	-0.50	IV	
P184	-0.50	0.25	-0.25	IV	
P185	-0.50	0.25	0.00	VI	
P186	-0.50	0.25	0.25	I	HP'
P187	-0.50	0.25	0.50	I	
P188	-0.50	0.25	0.75	I	
P189	-0.50	0.25	1.00	I	
P191	-0.50	0.50	-0.75	IV	
P193	-0.50	0.50	-0.25	IV	
P195	-0.50	0.50	0.25	I	
P197	-0.50	0.50	0.75	I	
P199	-0.50	0.75	-1.00	IV	
P200	-0.50	0.75	-0.75	IV	
P201	-0.50	0.75	-0.50	IV	
P202	-0.50	0.75	-0.25	IV	
P203	-0.50	0.75	0.00	VI	
P204	-0.50	0.75	0.25	I	
P205	-0.50	0.75	0.50	I	
P206	-0.50	0.75	0.75	I	
P207	-0.50	0.75	1.00	I	
P209	-0.50	1.00	-0.75	IV	
P211	-0.50	1.00	-0.25	IV	
P213	-0.50	1.00	0.25	I	
P215	-0.50	1.00	0.75	I	

Table S1. Artificial potentials for binary alphabets

ID	ϵ_{ii}	ϵ_{jj}	ϵ_{ij}	Type	Canonical
P217	-0.25	-0.25	-1.00	III	
P218	-0.25	-0.25	-0.75	III	
P219	-0.25	-0.25	-0.50	III	
P222	-0.25	-0.25	0.25	II	AB
P223	-0.25	-0.25	0.50	II	AB'
P224	-0.25	-0.25	0.75	II	
P225	-0.25	-0.25	1.00	II	
P226	-0.25	0.00	-1.00	VI	
P227	-0.25	0.00	-0.75	VI	
P228	-0.25	0.00	-0.50	VI	
P229	-0.25	0.00	-0.25	VI	
P230	-0.25	0.00	0.00	VI	HP
P231	-0.25	0.00	0.25	VI	
P232	-0.25	0.00	0.50	VI	
P233	-0.25	0.00	0.75	VI	
P234	-0.25	0.00	1.00	VI	
P235	-0.25	0.25	-1.00	IV	
P236	-0.25	0.25	-0.75	IV	
P237	-0.25	0.25	-0.50	IV	
P238	-0.25	0.25	-0.25	IV	
P239	-0.25	0.25	0.00	VI	
P240	-0.25	0.25	0.25	I	
P241	-0.25	0.25	0.50	I	
P242	-0.25	0.25	0.75	I	
P243	-0.25	0.25	1.00	I	
P244	-0.25	0.50	-1.00	IV	
P245	-0.25	0.50	-0.75	IV	
P246	-0.25	0.50	-0.50	IV	
P247	-0.25	0.50	-0.25	IV	
P248	-0.25	0.50	0.00	VI	
P249	-0.25	0.50	0.25	I	
P250	-0.25	0.50	0.50	I	
P251	-0.25	0.50	0.75	I	
P252	-0.25	0.50	1.00	I	
P253	-0.25	0.75	-1.00	IV	
P254	-0.25	0.75	-0.75	IV	
P255	-0.25	0.75	-0.50	IV	
P256	-0.25	0.75	-0.25	IV	

Table S1. Artificial potentials for binary alphabets

ID	ϵ_{ii}	ϵ_{jj}	ϵ_{ij}	Type	Canonical
P257	-0.25	0.75	0.00	VI	
P258	-0.25	0.75	0.25	I	
P259	-0.25	0.75	0.50	I	
P260	-0.25	0.75	0.75	I	
P261	-0.25	0.75	1.00	I	
P262	-0.25	1.00	-1.00	IV	
P263	-0.25	1.00	-0.75	IV	
P264	-0.25	1.00	-0.50	IV	
P265	-0.25	1.00	-0.25	IV	
P266	-0.25	1.00	0.00	VI	
P267	-0.25	1.00	0.25	I	
P268	-0.25	1.00	0.50	I	
P269	-0.25	1.00	0.75	I	
P270	-0.25	1.00	1.00	I	
P274	0.00	0.00	-0.25	VI	
P280	0.00	0.25	-1.00	VI	
P281	0.00	0.25	-0.75	VI	
P282	0.00	0.25	-0.50	VI	
P283	0.00	0.25	-0.25	VI	
P290	0.00	0.50	-0.75	VI	
P292	0.00	0.50	-0.25	VI	
P298	0.00	0.75	-1.00	VI	
P300	0.00	0.75	-0.50	VI	
P301	0.00	0.75	-0.25	VI	
P308	0.00	1.00	-0.75	VI	
P310	0.00	1.00	-0.25	VI	
P316	0.25	0.25	-1.00	V	
P317	0.25	0.25	-0.75	V	
P318	0.25	0.25	-0.50	V	AB'
P319	0.25	0.25	-0.25	V	AB
P325	0.25	0.50	-1.00	V	
P326	0.25	0.50	-0.75	V	
P327	0.25	0.50	-0.50	V	
P328	0.25	0.50	-0.25	V	
P334	0.25	0.75	-1.00	V	
P335	0.25	0.75	-0.75	V	
P336	0.25	0.75	-0.50	V	
P337	0.25	0.75	-0.25	V	

Table S1. Artificial potentials for binary alphabets					
ID	ϵ_{ii}	ϵ_{jj}	ϵ_{ij}	Type	Canonical
P343	0.25	1.00	-1.00	V	
P344	0.25	1.00	-0.75	V	
P345	0.25	1.00	-0.50	V	
P346	0.25	1.00	-0.25	V	
P353	0.50	0.50	-0.75	V	
P361	0.50	0.75	-1.00	V	
P362	0.50	0.75	-0.75	V	
P363	0.50	0.75	-0.50	V	
P364	0.50	0.75	-0.25	V	
P371	0.50	1.00	-0.75	V	
P373	0.50	1.00	-0.25	V	
P379	0.75	0.75	-1.00	V	
P388	0.75	1.00	-1.00	V	
P389	0.75	1.00	-0.75	V	
P390	0.75	1.00	-0.50	V	
P391	0.75	1.00	-0.25	V	
P398	1.00	1.00	-0.75	V	
P275	0.00	0.00	0.00	VII	

The raw data for each sequence-structure map in Table S1, can be downloaded at: www.santafe.edu/∼eferrada.

Table S2. Glossary of symbols and abbreviations

Symbol	Name
SEM	Simple Exact Model.
L18	SEM of polymer length 18 mer.
L	Length.
$\mathcal{A}$	Monomer alphabet.
α	Monomer alphabet size.
$\mathbf{U}$	Potential energy function.
$\mathcal{Q}_{\alpha}^{L}$	n-cube.
$\mathcal{G}$	Genotype space.
$\mathcal{P}$	Phenotype space.
$\mathcal{P}_u$	Set of encodable phenotypes.
n	Genotype space dimension.
s_i	Sequence.
c_i	Conformation.
h	Hamming distance.
E	Total stability.
g	Sequence degeneracy.
γ_k^i	Phenotypic diversity of sequence i in its k-neighborhood.
$\mathcal{S}$	Set of non-degenerate sequences.
$\mathcal{C}$	Set of accessible conformations.
ν	Non-degeneracy.
c	Encodability.
$\mathcal{F}$	Foldability.
m_{ij}	Mutational path between s_i and s_j.
D	Graph diameter.
$\mathcal{N}_G$	Networks of genotypes.
$\mathcal{X}_G$	Genotype component.
$\mathcal{N}_P$	Neutral set.
$\mathcal{Y}_P$	Neutral network.
C_{des}	$\mathcal{N}_P$ designability.
C_{neut}	$\mathcal{Y}_P$ neutrality.
λ	Sequence neutrality.
E_{ideal}	Ideal part of a potential.
E_{excess}	Excess part of a potential.
θ	Additivity of a potential.
O_x	Expected size of component x.
$F^k(d)$	Fraction of unique phenotypes in neighborhood at distance d.
$J_{ab}^{\mathcal{K}}$	Jaccard index.
